# In-depth mapping of protein localizations in whole tissue by micro-scaffold assisted spatial proteomics (MASP)

**DOI:** 10.1038/s41467-022-35367-2

**Published:** 2022-12-14

**Authors:** Min Ma, Shihan Huo, Ming Zhang, Shuo Qian, Xiaoyu Zhu, Jie Pu, Sailee Rasam, Chao Xue, Shichen Shen, Bo An, Jianmin Wang, Jun Qu

**Affiliations:** 1 Department of Pharmaceutical Sciences, SUNY at Buffalo, Buffalo, NY 14214 USA; 2 Roswell Park Comprehensive Cancer Center, Buffalo, NY 14203 USA; 3 New York State Center of Excellence in Bioinformatics & Life Sciences, Buffalo, NY 14203 USA; 4 Department of Biochemistry, Jacobs School of Medicine and Biomedical Sciences, SUNY at Buffalo, Buffalo, NY 14203 USA; 5 Department of Chemical and Biological Engineering, SUNY at Buffalo, Buffalo, NY 14214 USA; 6 Department of DMPK, Huiyu (Seacross) Pharmaceuticals Ltd, Chengdu, 610219 China

**Keywords:** Neuroscience, Analytical biochemistry, Imaging, Proteomics

## Abstract

Accurate, in-depth mapping of proteins on whole-tissue levels provides comprehensive insights into the spatially-organized regulatory processes/networks in tissues, but is challenging. Here we describe a micro-scaffold assisted spatial proteomics (MASP) strategy, based on spatially-resolved micro-compartmentalization of tissue using a 3D-printed micro-scaffold, capable of mapping thousands of proteins across a whole-tissue slice with excellent quantitative accuracy/precision. The pipeline includes robust tissue micro-compartmentalization with precisely-preserved spatial information, reproducible procurement and preparation of the micro-specimens, followed by sensitive LC-MS analysis and map generation by a MAsP app. The mapping accuracy was validated by comparing the MASP-generated maps of spiked-in peptides and brain-region-specific markers with known patterns, and by correlating the maps of the two protein components of the same heterodimer. The MASP was applied in mapping >5000 cerebral proteins in the mouse brain, encompassing numerous important brain markers, regulators, and transporters, where many of these proteins had not previously been mapped on the whole-tissue level.

## Abstract (web summary)

Accurate protein mapping on whole-tissue levels provides critical insights into diseases/therapies. Here, the authors described a novel spatial proteomics method, based on tissue compartmentalization using a 3D-printed micro-scaffold, generated thousands of protein maps across a whole-tissue slice.

## Introduction

Quantitative, proteome-level characterization of the often heterogeneous protein distributions on the whole-tissue level provides extensive insights into the spatially-organized regulatory processes/networks across all tissue regions, which is critical for a systemic understanding of biological functionality, disease mechanisms, and therapeutic effects. For example, heterogeneous distribution of protein drugs across tissues, is frequently responsible for compromised efficacy and/or undesirable side effects in clinical practice^1–3^. Characterization of the region-to-region variability in distribution of protein drugs as well as the drug targets and markers for efficacy/safety across the entire tissue, will afford highly valuable information to inform engineering and therapeutic efforts.

However, spatially-resolved, accurate mapping of the proteome across a whole tissue, represents a daunting challenge owing to the difficulties in sensitive and quantitative mapping of the numerous tissue proteins. For example, state-of-the-art immunoassay-based methods (e.g., multiplex-IHC) have generated amazing tissue mapping datasets, while falling short in a relatively low number of targets per mapping analysis (<40) and often require extensive validation owing to concerns about antibody specificity and reproducibility^4,5^. Matrix-assisted laser desorption/ionization mass spectrometry imaging (MALDI-MSI) has been tremendously successful in measuring the distribution of small-molecule markers in tissues^6,7^; however, mapping of proteins with MALDI-MSI, especially the low-abundance ones, remains exceedingly difficult. Recently, liquid chromatography-mass spectrometry (LC-MS)-based techniques demonstrated exceptional sensitivity for protein analysis^8–11^, which opens the possibility of spatially-resolved proteomics analysis by separating the tissue into micro-size, region-specific specimens, followed by LC-MS quantification. Towards this end, several works have been reported. A trail-blazing study cut voxels from tissue slides with a steel blade device and analyzed ~1000 proteins with spatial information. Imperfections include low spatial resolution (1000 × 1000 µm) and difficulty in precise spatially-resolved micro-sampling^12^. Another elegant strategy is laser-microdissection (LMD)-assisted NanoPOTS imaging, which was demonstrated in profiling the protein distribution across 24 micro-tissue areas^13^. Most recently, a powerful Deep Visual Proteomics strategy, which utilizes an artificial-intelligence-driven LMD technique to procure spatially-specific samples at single-cell or even subcellular levels^14^, was developed for spatial proteomics analysis.

These outstanding LMD-based methods have been successfully demonstrated in the survey of microscopic, focused tissue areas, but not feasible to measure protein maps on the whole-tissue level. The capacity of whole-tissue proteome mapping with deeper proteome coverage than the LMD-based methods would provide more extensive insights toward a deeper understanding of the region-specific biological regulations and drug effects^1–3^ and therefore is highly complementary to the LMD-based methods.

However, such a method has not been developed, largely because of the absence of a robust and reliable micro-sampling method that enables uniform compartmentalization of a whole-tissue slice while precisely preserving the spatial information.

**Fig. 1:**
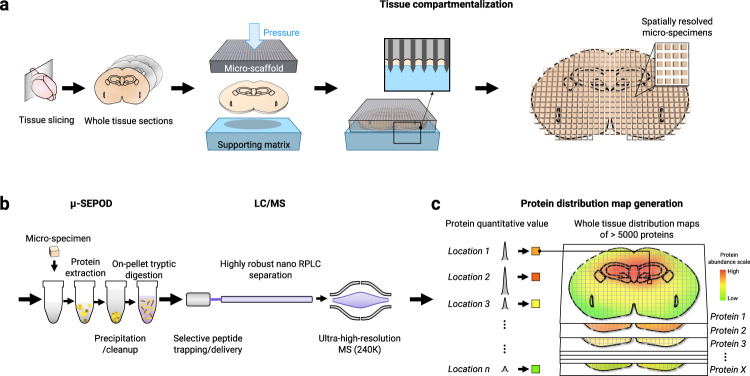
Scheme of the MASP strategy which enables accurate, quantitative, and in-depth protein mapping across a whole tissue slice. **a** Robust tissue micro-compartmentalization with precisely-preserved spatial information, by a 3D-printed micro-scaffold. **b** Efficient/reproducible preparation of the location-specific micro-specimens (micro-surfactant-aided extraction/precipitation/on-pellet digestion, µ-SEPOD) followed by robust/sensitive LC-MS analysis. **c** Protein mapping by the MAsP app, which matches the accurately-measured protein abundance to the corresponding location of each micro-specimen. The app also generates customizable protein maps and offers various post-processing functions such as clustering or correlation of protein distribution patterns (more details are in the Methods and Supplementary Notes). The protein abundance scale is from low (green) to high (red).

To address this important need and achieve sensitive, robust, and quantitative proteomics mapping covering all regions of a tissue slice, we devised a pipeline, micro-scaffold assisted spatial proteomics (MASP), capable of acquiring the distribution maps for thousands of proteins across a whole-tissue slice with excellent quantitative quality. The MASP consists of three components: first, robust and precise tissue micro-compartmentalization using a 3D-printed micro-scaffold (Fig. 1a); second, efficient and reproducible extraction, clean-up, and digestion of the location-specific micro-specimens followed by a sensitive/reproducible LC-MS analysis (Fig. 1b); third, generation of protein distribution maps with a MAsP app following accurate protein quantification^15,16^ (Fig. 1c). As a proof of concept, we showed an application of the MASP pipeline in mapping a mouse brain, where >5000 cerebral protein distribution maps, one for each protein, were obtained at the whole-tissue level. The reliability of the mapping technique has been extensively validated.

## Results

### Development and optimization of the MASP strategy

**Fig. 2:**
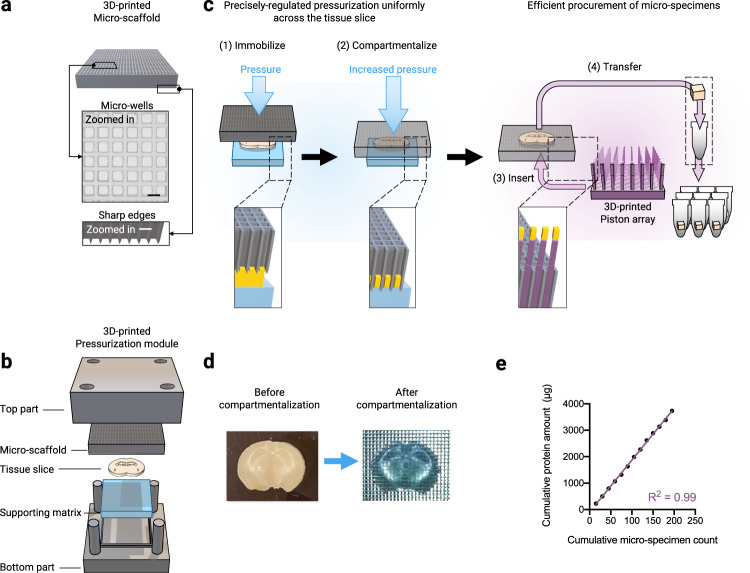
The workflow for precise micro-compartmentalization of the tissue and the procurement of spatially resolved micro-specimens. **a** The 3D-printed micro-scaffold with narrow edges to achieve precise tissue compartmentalization (scale bar: 400 µm). **b** The assembly of the 3D-printed pressurization module with the stack of micro-scaffold, tissue slice, and the supporting matrix (from upper to lower), to enable precisely-regulated and uniform pressurization across the entire tissue slice. The detailed pressurization procedure is shown in Supplementary Movie 1. **c** The schematic procedure for robust tissue micro-compartmentalization followed by efficient procurement of micro-specimens. A precisely-regulated pressure is applied on the micro-scaffold, which uniformly immobilizes (1) and then compartmentalizes the tissue slice with elevated pressure (2). Next, a 3D-printed piston array with breakable pistons is inserted into the micro-scaffold to push the micro-specimens out of the micro-wells, detailed in Supplementary Fig. 4 (3). Then, the micro-specimens are transferred to sample tubes (4). **d** The photos of a brain tissue slice before (left) and after (right) the compartmentalization. The entire tissue slice was uniformly and completely compartmentalized without any left-over tissue, while the spatial information was faithfully maintained. **e** Exceptional linearity between cumulative protein amounts versus cumulative numbers of micro-specimens, indicating excellent reproducibility and robustness of both micro-compartmentalization and sample preparation, which sets a solid foundation for reliable protein mapping.

Each of the components of the MASP was rigorously optimized to achieve accurate, quantitative protein mapping on the whole-tissue level. Firstly, robust and uniform micro-compartmentalization of a tissue slice while precisely preserving the original spatial information, is the very foundation for accurate mapping. In MASP, this was achieved by well-regulated pressurization of a 3D-printed micro-scaffold, which contains numerous precisely-spaced cubicles, against a tissue slice (Fig. 1a). The rationale and workflow of the micro-compartmentalization procedure are illustrated in Fig. 2.

To avoid tissue distortion while attaining complete separation of individual micro-specimens, several measures were taken (details in Supplementary Notes): (i) based on extensive evaluations on resolution, throughput, and properties of the materials, we chose to fabricate polymer-based micro-scaffolds. The example shown in Fig. 2a and Supplementary Fig. 1a consists of 900 precisely-spaced micro-wells with a 400-μm spatial resolution. When pressurizing the micro-scaffold against a tissue slice, the narrow edges of the micro-wells simultaneously immobilize all regions of a tissue slice to prevent tissue distortion, and then with increased pressure, cut through the tissue into a layer of supporting matrix (Fig. 2b, c) to allow robust tissue compartmentalization with completely separated micro-specimens; (ii) we found placing a layer of proper supporting matrix underneath the tissue during the pressurization step (Fig. 2b), is critical to achieve high-quality micro-compartmentalization. The ideal material should have sufficient elasticity so that it can protrude into the micro-wells under pressurization to facilitate effective shearing of tissue against the narrow edges of the micro-scaffold, thus fully separating individual tissue specimens (Supplementary Fig. 2). The material should also be sufficiently strong to sustain the high pressure that is required for micro-compartmentalization, and meanwhile be rupture-resistant, and introduce no contaminating proteins or detrimental polymers. After carefully assessing various candidates that are starch-, PVC- or silicon-based, we selected PDMS because it provides optimal support for complete tissue compartmentalization without protein contaminations; (iii) our preliminary study indicated that well-regulated pressurization uniformly across all the regions of the tissue slice is essential to attain robust and reproducible micro-compartmentalization. To achieve this goal, we devised a 3D-printed pressurization module, which encloses the stack of micro-scaffold, a tissue slice, and the supporting matrix (in the upper-to-lower order, Fig. 2b, Supplementary Fig. 1b, c). The precisely-regulated pressure is applied on the top part of the module, to firstly immobilize and then completely compartmentalize the tissue slice (Fig. 2c, Supplementary Movie 1).

Under the optimized conditions (Supplementary Notes, Supplementary Fig. 3), the entire tissue slice was uniformly and completely compartmentalized without any left-over tissue, while the spatial information was faithfully maintained, as exemplified in Fig. 2d. To enable efficient, high-throughput transfer of micro-specimens out of the scaffold, we devised a series of 3D-printed piston arrays (a total of 16 arrays with staggered positions were designed to cover one micro-scaffold) with breakable pistons to rapidly procure the tissue micro-specimens (Supplementary Notes, Supplementary Fig. 4a). On each array, the pistons match one out of every adjacent four micro-wells in both horizontal and vertical directions. The pistons were chamfered and therefore breakable, so that the upper part of the pistons carrying micro-specimens can be easily clipped off and transferred into low-protein-binding tubes for subsequent sample preparation (Fig. 2c, Supplementary Fig. 4b). Finally, we found that ~70–80% humidity, −5 to 0 °C temperature, and free-of-oxygen are all important to successfully transfer the tissue micro-specimens. Therefore, the process was performed in an enclosed chamber that regulates the above conditions. With the approach, 100% success rate in the procurement of high-quality micro-specimens from tissue slices has been achieved, compared to ~52% when operated in the open air (Supplementary Notes, Supplementary Fig. 5).

Secondly, efficient/reproducible sample preparation and LC-MS analysis, robustly across all micro-specimens, is another important prerequisite for reliable mapping of tissue proteins. Previously, we described a SEPOD strategy, which employs a strong-detergent cocktail and provides near-complete tissue protein extraction, thorough cleanup of samples, and exceptional peptide recovery^17^. Here, we modified and optimized the procedure to attain efficient preparation, reproducibly across all micro-specimens (termed µ-SEPOD, Fig. 1b). Details are in Supplementary Notes and Supplementary Fig. 6. As exemplified in Fig. 2e, the cumulative recovered protein amounts versus the number of micro-specimens showed exceptional linearity (R^2^ = 0.99) across a tissue slice, indicating excellent reproducibility and robustness of both micro-compartmentalization and sample preparation, which sets a solid foundation for reliable protein mapping. For LC-MS analysis, a trapping nano-flow LC system capable of extensive and robust analysis of large cohorts^18^ was employed. Moreover, the combination of ultra-high-resolution MS1(240 K FWHM@*m/z* = 200) and UHR-IonStar^15,16^ provides sensitive and accurate protein quantification, including the low-abundance ones (Supplementary Notes). All samples were prepared/analyzed in a random order to avoid analytical artifacts.

Thirdly, to generate and further analyze protein distribution maps in tissues, we developed an R-based spatial mapping app (MAsP, v 1.0, available at https://github.com/JunQu-Lab/MAsP Fig. 1c) with a graphical user interface (GUI). The module generates customizable protein maps and offers various post-processing functions such as clustering or correlation of protein distribution patterns (Supplementary Notes, Supplementary Fig. 7). The MAsP app constructs customized protein distribution maps based on the spatial coordinates and protein abundances of individual micro-specimens. The app also offers several functions for further analysis of the generated maps. For example, the “Map clustering” function was designed to group protein maps with similar distribution patterns, using a density-based clustering algorithm for image processing^19^. Furthermore, the discovery of proteins with positively- or negatively-correlated distribution patterns provides valuable information on spatially organized biological processes. To identify these correlations, in MAsP, we also devised a “Map correlation” module to discover the proteins that have distribution maps correlating with the map of a protein of interest, based on either Pearson correlation coefficient or cosine similarity.

### Application of the MASP strategy in the mapping of the cerebral distribution of >5000 proteins

**Fig. 3:**
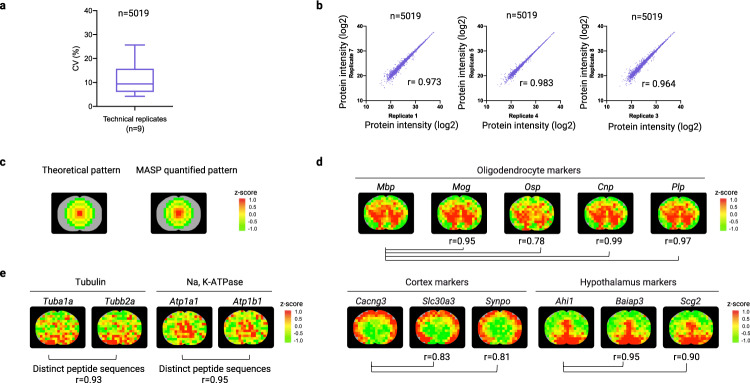
The high reproducibility and accuracy of the MASP pipeline. **a** Intra-group CV% of the quantitative values of 5019 quantified proteins among sample preparation replicates of a pooled quality control (QC) sample (analyzed once every 20 micro-specimens, *N* = 9). The box shows the 25th to 75th percentile range with the median indicated by a horizontal line. Whiskers extend to the 10th and 90th percentile range. **b** The correlation of quantitative values of 5019 quantified proteins between randomly-selected runs of the QC sample. **c** The agreement between the theoretical distribution and MASP-acquired map of strategically spiked non-endogenous peptides. **d** The maps of oligodendrocyte markers, *Mbp*, *Mog*, *Osp, Cnp, and Plp*, agreed well with the expected patterns and showed highly correlated distributions (Pearson correlation *r* = 0.78–0.99) (upper); the distribution maps of proteins highly-expressed in the cortex or hypothalamus (lower). **e** High correlation of the maps of the two distinct proteins that form the same heterodimeric protein complex, including the heterodimers of the tubulins and Na, K-ATPases. The z-score color scale for **c**–**e** is from −1.0 (green) to 1.0 (red). Source data are provided as a Source Data file.

The MASP was applied to measure the whole-tissue distribution of the proteins in the brain of a healthy mouse. Under the stringent cutoff thresholds for identification and quantification (details in Methods), 5019 unique proteins were quantified and mapped in the brain slice (anatomical position is shown in Supplementary Fig. 8). Complete distribution maps were obtained for 91.2% of these proteins (i.e., protein quantified in all locations of the brain slice, *N* = 208 micro-specimens), and 98.2% of these proteins were mapped in at least 95% of all locations. A list of all 5019 quantified proteins and their log2 abundances with spatial locations are in Supplementary Data 1. The detailed spatial locations are shown in Supplementary Data 2. To evaluate the quantitative quality, aliquots of 40 randomly-selected micro-specimens were pooled to create a QC sample, which was prepared and then analyzed between the analysis of every 20 micro-specimens. The quantitative method achieved low intra-group CV for protein abundance values (median CV = 9.4%, *N* = 9, Fig. 3a) and superb reproducibility for protein quantification (Pearson correlation *r* = 0.964–0.983 between randomly-selected sample preparation replicates of QC samples, Fig. 3b). The correlations of all QC samples were shown in Supplementary Fig. 9.

### Validation of the mapping accuracy of MASP

The mapping accuracy of MASP was validated in three ways.

First, we spiked low, strategically-varied levels of non-endogenous, synthetic peptides into the micro-specimens collected at different locations (Supplementary Notes, Supplementary Fig. 10), so that a designed pattern would show up in the map of these peptides if the MASP achieved accurate mapping. We observed that MASP faithfully produced the expected patterns with excellent quantitative accuracy (mean quantitative error% = 12.9%, Fig. 3c).

Second, we investigated the MASP-generated maps of the proteins with previously-known distribution patterns in the brain. Though for the vast majority of cerebral proteins, the whole-tissue level distributions remain unknown owing to technical limitations, the intra-brain distributions of a small number of markers have been reported, such as the markers of oligodendrocytes and a handful of brain-region-specific markers^20,21^. It turned out that MASP correctly recapitulated the expected, region-specific distributions of these proteins, with excellent correlations among them. For example, the MASP-generated map of myelin basic protein (*Mbp)*, a well-known oligodendrocyte marker, agreed well with its previously-reported distributions^20^ (Fig. 3d). We further compared the map of *Mbp* with four other oligodendrocyte markers^22,23^: myelin oligodendrocyte glycoprotein (*Mog*), oligodendrocyte-specific protein (*Osp*), 2’,3’-cyclic-nucleotide 3’-phosphodiesterase (*Cnp*), and myelin proteolipid protein (*Plp*). As shown in Fig. 3d, the MASP-generated maps of these markers are consistent with the map of *Mbp* (Pearson correlation *r* values of 0.95, 0.78, 0.99, and 0.97, respectively). By comparison, the median *r* value for correlating *Mbp* with all other proteins was only 0.05; moreover, using a stringent Bonferroni correction method, it was found a Pearson correlation *r* > 0.3 is significant (*p* < 0.05). Therefore, the correlations of these maps are significant, suggesting reliable mapping by MASP.

We also surveyed the maps of some protein markers that are known to be enriched in other brain anatomical regions^22,24^. Figure 3d showed that MASP correctly recapitulated the expected protein distributions in various anatomical regions such as the cortex (e.g., *Cacng3, Slc30a3, Synpo*) and hypothalamus (e.g., *Ahi1, Baiap3, Scg2*) with high correlations among the markers (Pearson’s *r* = 0.81–0.95). More examples of regionally enriched proteins are shown in Supplementary Fig. 11, where the MASP-generated protein distribution maps exhibited patterns consistent with those reported in the literature.

Thirdly, we examined the mapping accuracy of MASP by correlating the spatial distributions of the two distinct proteins that form the same heterodimeric protein complex. If the MASP method was reliable, the maps of the two components of a heterodimer that were independently acquired by MASP, should show correlated distribution patterns. Here only the heterodimers that meet the following two criteria were chosen to validate the MASP technique: (i) the two proteins in the heterodimer must have distinct, non-overlapping sequences, because the shared peptides between the two proteins might lead to overestimation of mapping accuracy; and (ii) the two protein components should exist mainly in the heterodimer form so that most of these two proteins are co-located. For instance, in cells, tubulin-α (*Tuba1a*) and tubulin-β (*Tubb2a*), two proteins with distinct sequences, mostly exist in the form of a 1:1 heterodimer^25^. Consequently, the spatial distributions of the tubulin α and β should be correlated if the mapping by MASP is accurate. Indeed, an excellent correlation (Pearson’s *r* = 0.93) between the maps of tubulin-α vs. tubulin-β was observed (Fig. 3e). Another example is that the individually-acquired maps of the heterodimeric sodium/potassium-ATPase α (*Atp1a1*) and β (*Atp1b1*)^26^ are highly correlated (Pearson’s *r* = 0.95, Fig. 3e).

### MASP demonstrated the potential to produce a valuable resource of whole-tissue protein maps and revealed landscapes of spatially-organized signaling pathways and biological functions

**Fig. 4:**
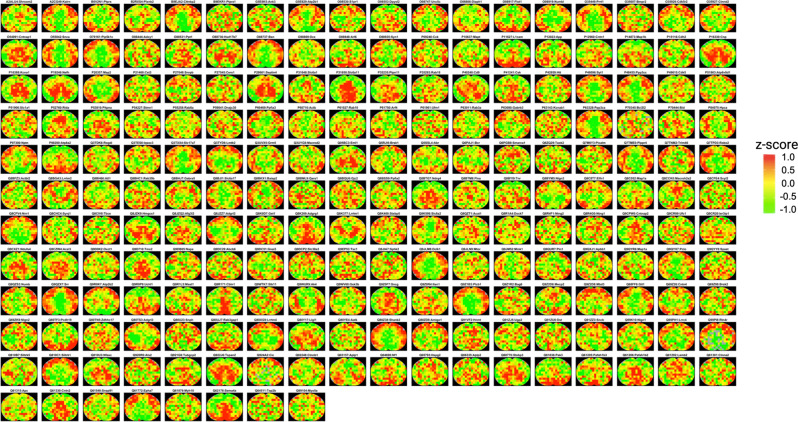
Representative examples showing MASP’s potential in providing insights into spatially-organized biological processes. The spatial distribution maps of the proteins involved in developmental processes in the brain, including brain development (GO:0007420), synapse organization (GO:0050808), and axonogenesis (GO:0007409). The z-score color scale is from −1.0 (green) to 1.0 (red).

The >5000 cerebral protein maps by MASP, mapped on the whole-tissue level, offered a unique resource comprising the intra-brain distribution maps for numerous proteins. The results revealed the considerable region-to-region variability for a large portion of brain proteins, which may extend our understanding of the spatially-resolved biological functions of different brain regions. We have mapped numerous brain proteins, for example, ~94% and ~89% of all the proteins with the DAVID annotation of “hippocampus” and “brain cortex” were respectively mapped. For instance, the MASP-generated maps of the proteins that are involved in the brain developmental processes are shown in Fig. 4.

One unique advantage of MASP is that it generates whole-tissue protein distribution maps for many important players in various signaling networks and pathways, which affords the potential to provide a panoramic insight into the spatially-organized biological functions, as illustrated in Supplementary Data 3 (KEGG pathways) and Supplementary Data 4 (Gene Ontology Biological Processes). Here, as a few examples among many, we discuss the potential of MASP in the characterization of the spatially-organized pathways involved in synapses and neurodegenerative diseases, and in the whole-tissue mapping of important neurotransmitters and drug transporters in the brain.

**Fig. 5:**
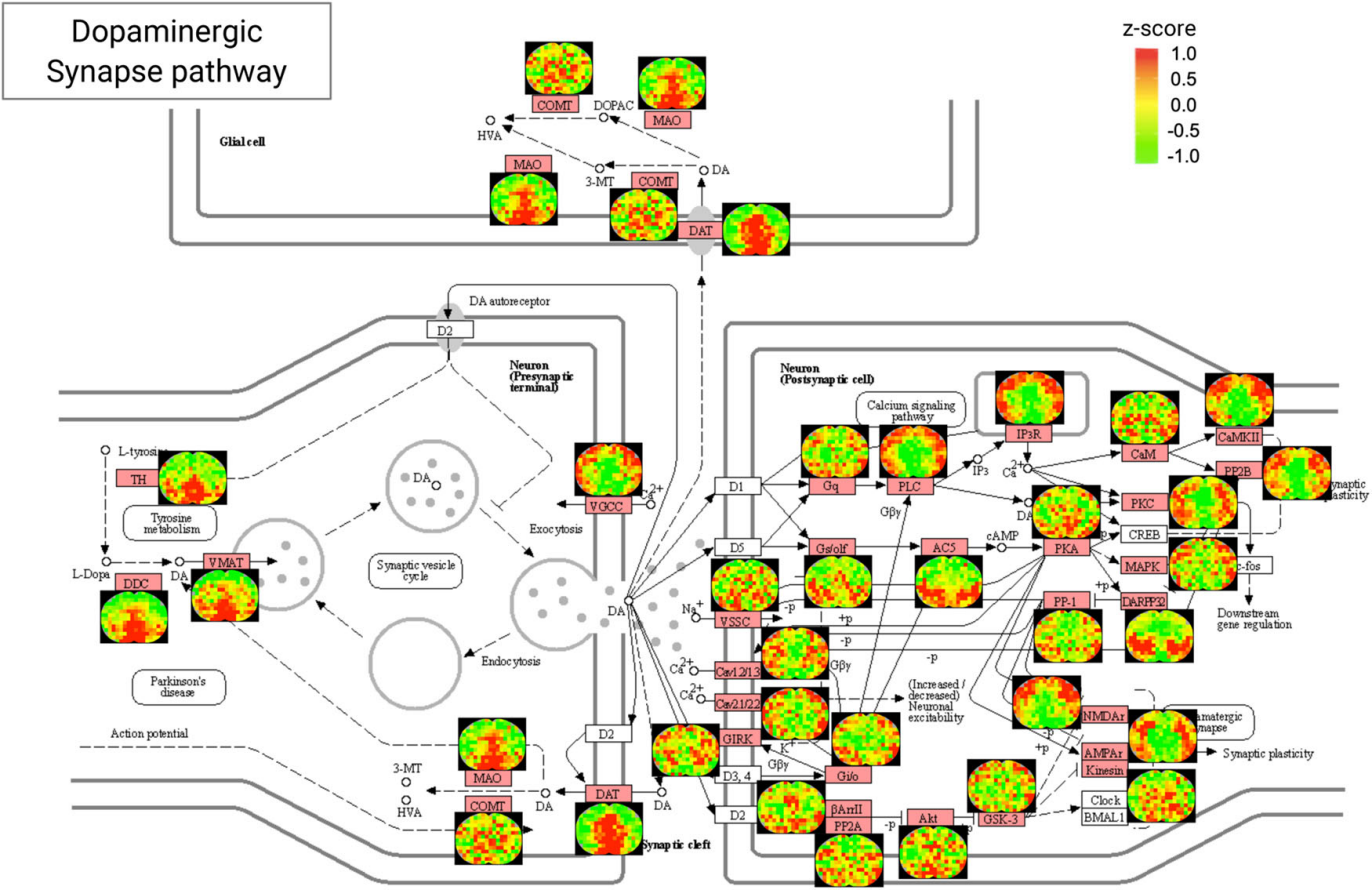
Cerebral distribution maps of key proteins involved in the KEGG pathway of the dopaminergic synapse acquired by MASP. The z-score color scale is from −1.0 (green) to 1.0 (red). The red boxes on the KEGG pathway plots denote the proteins quantified by MASP.

The first example includes the maps of proteins that are components of important synaptic pathways. Mapping the intra-brain distribution of the key components of synaptic pathways, which are implicated in a wide range of neurodegenerative diseases such as Alzheimer’s disease (AD) and Parkinson’s disease (PD) as well as cognitive function and aging^27^, would provide valuable insights into the diseases and therapies. With MASP, we were able to achieve remarkable coverage in important synaptic pathways: ~76% protein components were mapped for glutamatergic synapse pathway, ~64% for dopaminergic synapse pathway, ~75% for GABAergic synapse pathway, ~57% for cholinergic synapse pathway, and ~43% for serotonergic synapse pathway (Supplementary Data 3). The mapped components in the dopaminergic synapse pathway, which are closely associated with the regulation of motor function in both PD and AD^28^, are shown in Fig. 5. Glutamate is the most common neurotransmitter in the brain and glutamatergic neurons, which is important for multiple brain functions and synaptic plasticity^27^. The maps of key players in the glutamatergic synapse pathway are shown in Supplementary Fig. 12a. The maps of regulators in the GABAergic synapse pathway, cholinergic synapse pathway, and serotonergic synapse pathway are shown in Supplementary Fig. 12b–d.

**Fig. 6:**
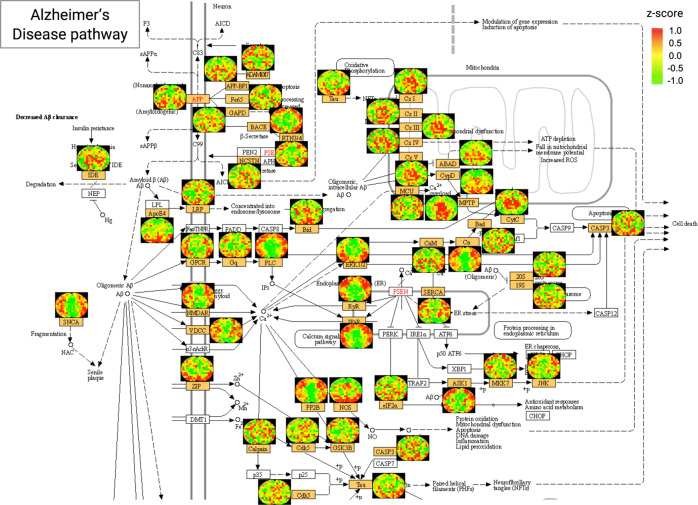
Cerebral distribution maps of key proteins involved in the KEGG pathway of Alzheimer’s disease acquired by MASP. The z-score color scale is from −1.0 (green) to 1.0 (red). The orange boxes on the KEGG pathway plots denote the proteins quantified by MASP.

The second example involves important neurodegenerative disease pathways. The underlying molecular mechanisms of many neurodegenerative diseases such as AD, PD, Huntington’s disease (HD) and prion diseases are poorly understood^28,29^. Investigation of the cerebral distribution of the key proteins involved in these disease pathways can provide valuable insights that may facilitate the elucidation of the disease mechanisms. In this study, MASP was able to map most of the proteins in these pathways, providing an extensive view of the intra-brain distribution of key players in these diseases. For example, MASP generated the maps for ~78% of the proteins involved in the PD pathway, ~71% of the proteins in HD pathway, ~61% of the proteins in the AD pathway (Fig. 6) and ~74% of proteins in the prion disease pathway (Supplementary Data 3). Among these, important proteins that are known to aggregate in some neurodegenerative diseases were mapped in the brain, such as huntingtin (aggregation in HD), amyloid-beta in AD, alpha-synuclein in PD, and prion protein in prion diseases.

**Fig. 7:**
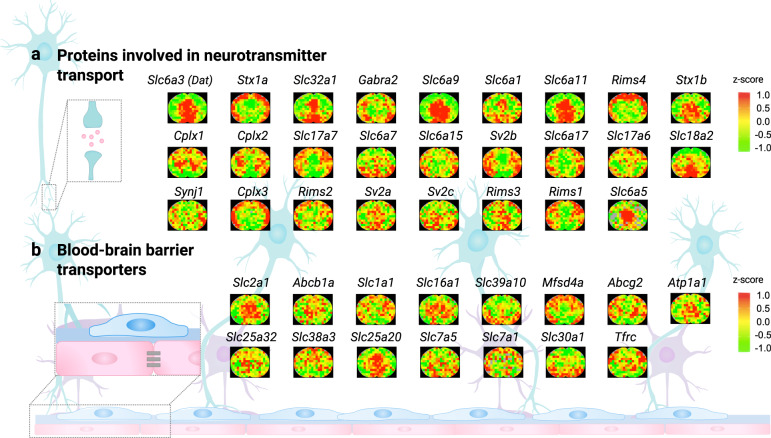
The maps of proteins involved in neurotransmitter transport and transporters expressed on the blood-brain barrier acquired by MASP. **a** The spatial distribution maps of proteins involved in the neurotransmitter transport (GO:0006836). **b** The spatial distribution maps of the important drug transporters expressed on the blood-brain barrier. The z-score color scale for **a**, **b** is from −1.0 (green) to 1.0 (red).

The third example encompasses the maps of proteins associated with neurotransmitter transport. Proteins involved in neurotransmitter transport mediate the uptake/efflux of neurotransmitters and thus shape the communication between the neurons. Because of their fundamental roles in maintaining the physiological functions of the brain, these proteins are often regarded as potential therapeutic targets for a spectrum of CNS disorders^30^. Mapping the spatial distribution of the proteins involved in neurotransmitter transport could provide the landscape of region-specific neurotransmitter transporting activities and may facilitate the evaluation of potential drug targets. In this study, the MASP acquired cerebral distributions of 26 proteins involved in neurotransmitter transport (Supplementary Data 4, distribution maps are shown in Fig. 7a).

The last example is the mapping of proteins associated with the blood-brain barrier (BBB). The BBB, which acts as a selective blood-brain interface, has been of high interest in the study of brain functions, disease mechanisms and therapies^31^. Particularly, as the BBB impedes the delivery of most drugs to the brain, the transporters carrying drugs or other important molecules across the BBB are highly critical for therapeutic efforts. Therefore, measuring the cerebral distributions of these transporters affords essential information for drug design and optimization of drug delivery^32^. Figure 7b showed the distribution maps of 15 representative transporters acquired by MASP, which encompass all known families of transporters across the BBB^33^, including ATP-driven efflux pumps (*Abcb1a, Abcg2, Atp1a1*), carrier-mediated transport proteins (*Slc1a1, Slc2a1, Slc16a1, Slc39a10, Slc25a32, Slc38a3, Slc25a20, Slc7a5, Slc7a1, Slc30a1*), receptor-mediated transporter proteins (*Tfrc*), and the endothelial facilitator superfamily (*Mfsd4a*).

## Discussion

With MASP, in-depth and accurate spatially-resolved proteomic mapping on the whole-tissue level is achieved. This study generated a valuable dataset allowing the users to explore the cerebral distribution maps of >5000 unique proteins, which revealed the prevalent region-to-region heterogeneity in the distributions of proteins across the brain. Such resource will not only expand our knowledge of spatially-resolved brain biology and functions, but also provides the basis to inform research concerning brain disease and therapy.

The whole-tissue mapping capacity by MASP is complementary to the spatial proteomics strategies based on single-cell-proteomics or LMD, which enables comprehensive investigations of biologically and pharmaceutically meaningful region-to-region variations, as well as the integration of functional/pharmaceutical information with spatial information. Consequently, the method will markedly facilitate the efforts toward the understanding of the spatially-organized biological regulations responsible for disease mechanisms and drug actions. For example, with MASP, one can investigate in what way a protein drug, its targets, and the efficacy/safety markers are spatially co-regulated in tissues, which could profoundly advance our understanding of how drug-induced biological cascades cooperate spatially to give rise to drug effects and side effects.

In MASP, the micro-compartmentalization and associated techniques are robust and versatile, which can be easily customized for different tissue types, and can be conjugated to any sample preparation, LC-MS, and protein quantification pipelines (e.g., multiplexed isotope-labeling, DIA, etc.), as long as an accurate, reproducible protein quantification across the micro-specimens is achieved. Future developments of MASP are ongoing in our labs, which include: (i) micro-scaffolds with higher spatial resolution, by taking advantage of the rapid advancements of the 3D-printing technologies; (ii) improvement of throughput on sample preparation and LC-MS analysis, e.g., automatic sample preparation with techniques such as SP3 magnetic beads^34^ and multiplexed analysis of micro-specimens with labeling techniques such as TMTpro^35^; (iii) expand the applications of MASP beyond proteomics such as mapping of target markers of interest or post-translational modifications, and simultaneous investigation of spatially-resolved metabolomics and proteomics in the same set of micro-specimens, using a multi-omics analysis system we described previously^36^.

## Methods

### Materials and reagents

Acetonitrile (ACN), Acetone, Formic Acid (FA), Methanol, Sodium dodecyl sulfate (SDS), and Sodium chloride (NaCl) were purchased from Fisher Scientific (MA, USA). Micro Bicinchoninic acid (BCA) protein assay was purchased from Thermo Fisher Scientific (CA, USA), Sodium deoxycholate, IGEPAL CA-630, Iodoacetamide (IAM), Trypsin (Proteomics grade), and cOmplete^TM^ Mini EDTA-free Protease Inhibitor were acquired from Sigma-Aldrich (MO, USA). Dithiothreitol (DTT) was obtained from Cytiva (MA, USA). Tris was purchased from MP Biochemicals (OH, USA). Protein Lobind tubes (i.e., low-protein-binding tubes) were purchased from Eppendorf (Hamburg, Germany).

### Fabrication of 3D-printed devices for micro-scaffold-assisted tissue compartmentalization and procurement

All 3D-printed devices are designed using Autodesk Fusion 360 software (Autodesk Inc., USA). The micro-scaffold, pressurization module, and piston arrays were printed in-house by the Advanced-DLP technique (LED wavelength of 385 nm) using a D4Kpro 3D desktop printer (EnvisionTEC, Germany), with resolutions of 25 µm and 1 µm for the X-Y-axes and the Z-axis, respectively. The printer was calibrated following the manufacturer’s protocol. The layer thickness was set to 25 µm and the printing build style was provided by the manufacturer and was specific to the printing material. We selected HTM140V2 (EnvisionTEC, Germany) resin owing to its high-resolution printing capability, excellent strength (Tensile Strength of 56 MPa), exceptional heat resistance (Heat Deflection Temperature up to 140 °C), and smooth surface finishing of the printed items. For curing, the micro-scaffold, pressurization module, and piston arrays were rinsed with 100% methanol, dried up by N_2_ gas, then cured by ultraviolet (UV) at a wavelength of 405 nm for 4 min. The computer-aided design (CAD) files (.stl) for the micro-scaffold and pressurization module are provided as Source Data files.

#### The 3D-printed micro-scaffold

The micro-scaffold contains an array of precisely spaced micro-wells. In the example shown in Fig. 2a, a micro-scaffold containing 30 × 30 wells was fabricated. The dimension for each well is 400 × 400 µm. Each micro-well has narrower edges at the bottom, which is designed to effectively immobilize the tissue slice upon the initial contact and thereby faithfully preserve spatial information (Fig. 2a in the zoomed-in panel, Supplementary Fig. 1a).

#### The 3D-printed pressurization module

As shown in Fig. 2b, and Supplementary Movie 1, the pressurization module includes two parts that together enclose the stack of the micro-scaffold, a slice of tissue, and a layer of supporting matrix. The regulated pressure is applied on the top part of the module, which transduces the pressure evenly to the micro-scaffold to achieve a uniform micro-compartmentalization. The module is carefully optimized and tested to ensure precise fitting and leveling of these components. The design with the detailed parameters and the actual 3D-printed product is shown in Supplementary Fig. 1b, c.

#### The 3D-printed piston arrays for high-throughput transfer of compartmentalized tissue micro-specimens out of the micro-scaffold

The design of a series of 3D-printed, single-use piston arrays, which fits the corresponding micro-scaffold shown in Fig. 2a, is exemplified in Supplementary Fig 4. When inserted into the micro-wells, the pistons push individual micro-specimens out of the micro-wells. The pistons are chamfered at 3.95 mm from the bottom to render them breakable so that the tips of the pistons carrying tissue specimens are readily clipped into low-protein-binding tubes. To allow easy clipping, sixteen different piston arrays with staggered piston positions were fabricated: on each array, there is one piston matching one out of every four micro-wells in both horizontal and vertical directions (Supplementary Fig. 4). Sequentially using the set of the arrays covers all of the wells on the micro-scaffold.

### The polydimethylsiloxane (PDMS) supporting matrix

The polydimethylsiloxane (PDMS) supporting matrix was prepared using SYLGARD™ 184 Silicone Elastomer (DOW Inc, USA) according to the manufacturer’s protocol (https://www.dow.com/en-us/pdp.sylgard-184-silicone-elastomer-kit.01064291z.html). Briefly, the mixture of Base Part A and Curing Agent Part B (10:1 v/v) was cured at 40 °C for 12 h. The resulting PDMS block was cut into 1.3 × 1.3 × 1 cm sheets to serve as the supporting matrix during tissue compartmentalization (more details in Supplementary Notes).

### The chamber with regulated conditions to enable successful procurement of tissue micro-specimens

To achieve the desired conditions for the micro-sampling process, we devised an enclosed chamber using clear acrylic sheet glass which holds a 70 L volume (Supplementary Fig. 5). The chamber is equipped with multiple LED lights to enable a shadowless illumination of the operation areas, a dry ice container, temperature/humidity sensors as well as controlled ventilating fans, and a humidifier. The conditions for micro-compartmentalization and micro-specimen transferring are regulated as the following: −5 to 0 °C, ~70-80% humidity, and the chamber is filled with CO_2_ to displace air.

### The tissue sectioning, micro-compartmentalization, and micro-specimen procurement

All animal experiments were performed according to the protocols approved by the Roswell Park Institutional Animal Care and Use Committee (IACUC). An eight-week-old male healthy Swiss Webster (CFW) mouse was anesthetized by isoflurane inhalation and then perfused with 15 mL heparinized saline^37^. The mouse brain was harvested and rinsed with saline, and then sectioned into a series of 1-mm-thick coronal slices using steel blades with an Adult Mouse Brain Slicer Matrix (BSMAS001-1, Zivic Instruments, USA). The slice at −1 to −2 mm from bregma was used for MASP mapping. The anatomical coordinates of the slice are shown in Supplementary Fig. 8. The brain slice was mounted on the supporting matrix and then pre-froze at −80 °C for ~30 min before compartmentalization (Supplementary Fig. 3).

The frozen slice was then compartmentalized into spatially-resolved micro-specimens using the 3D-printed micro-scaffold, following these steps: first, the slice was mounted on a layer of PDMS supporting matrix which was securely settled into the holder on the bottom part of the pressurization module, followed by mounting a pre-cooled (in −20 °C) micro-scaffold on the tissue slice with the sharp edge facing the tissue. Second, the top part of the pressurization module (pre-cooled) is assembled to enclose the stack, and then pressure is applied to the top plate of the pressurization module via a steel press machine (VEVOR, USA) with a well-controlled pressure, and the pressure was monitored by a Fafeicy force-sensing resistor (Adafruit, USA) connected to a calibrated digital ohmmeter (Cen-Tech, USA) (Supplementary Movie 1). The initial pressure (~3–5 kg) was applied to immobilize the tissue, after holding for 10 s, the pressure was then increased to ~10–15 kg to finalize the micro-compartmentalization.

As described above, the micro-specimens were collected sequentially by the series of 16 piston arrays at −5 to 0 °C, ~70–80% humidity, saturated CO_2_ in the specimen-procurement chamber and then transferred into the low-protein-binding tubes that are pre-marked with the corresponding spatial coordinates.

### The microscale Surfactant Cocktail-Aided Extraction/Precipitation/On-Pellet Digestion (µ-SEPOD) for preparation of the micro-specimens

The sample preparation protocol was optimized for the micro-specimens by modifying the Surfactant Cocktail-Aided Extraction/Precipitation/On-Pellet Digestion (SEPOD) protocol^17,36^. All procedures were performed on ice unless indicated otherwise. For exhaustive protein extraction, 30 µL of surfactant cocktail buffer (50 mM Tris-FA pH = 8.5, 150 mM NaCl, 0.5% sodium deoxycholate, 2% IGEPAL CA-630, 2% SDS, cOmplete^TM^ Mini, EDTA-free Protease Inhibitor Tablets) was added into the tube of each tissue micro-specimens. The samples were sonicated in a water bath for 15 min at room temperature and then incubated at 4 °C overnight. The tissue lysates were vortexed and then centrifuged at 20,000 g (4 °C, 30 min). The supernatant was carefully collected into the low-protein-binding tubes, and the remaining cell debris along with the snapped tip of the pistons was discarded. The total protein concentration of each sample was measured using the Micro BCA Protein Assay. For each micro-specimen, 10 µg of proteins were reduced by 10 mM DTT (56 °C, 30 min), then alkylated by 25 mM IAM (37 °C for 30 min, in darkness) in a Thermomixer (Eppendorf, Germany) at 550 rpm. After adding 6× volumes of chilled acetone (−20 °C), the samples were placed at −20 °C for 3 h for protein precipitation. Then, the samples were centrifuged using 20,000 g at 4 °C for 30 min, the supernatant was discarded, and the pelleted protein was carefully washed with 300 µL of methanol, then resuspended into 28 µL of 50 mM Tris-FA (pH = 8.5). Activated trypsin of 2 µL (0.25 µg/µL) was added at an enzyme-to-substrate ratio of 1:20 (w/w); and the tryptic digestion was performed in the Thermomixer at 37 °C, 550 rpm for 6 h. The digestion was terminated by adding FA to 1% (v/v) final concentration followed by centrifugation at 20,000 g, 4 °C for 30 min. The supernatant was transferred to autosampler vials for the LC-MS analysis.

In parallel, a quality control (QC) sample was prepared to monitor the technical variations. The QC sample contains 200 µg of total proteins that were pooled from 40 randomly selected micro-specimens. The QC sample was aliquoted and digested following the protocol described above.

### Liquid chromatography-mass spectrometry

All spatially-specific micro-specimens were analyzed in a randomized order. The QC sample was injected in between every twenty runs, to monitor the analytical performance. A unique trapping-nano LC-high resolution MS system was used^15,18^. The LC-MS system consists of an UltiMate 3000 gradient Micro LC system, an UltiMate 3000 Nano LC system, a WPS-3000 autosampler, and an Orbitrap Fusion Lumos Tribrid Mass Spectrometer (Thermo Fisher Scientific, USA). The peptides derived from ~2.5 µg proteins were firstly delivered onto a trapping column (5 × 300 µm I.D.) at a flow rate of 10 µL/min with 1% B, to selectively remove the hydrophilic and hydrophobic matrix components. Then the peptides were loaded onto a nano-LC column (65 cm × 75 µm I.D., 2.5 µm PepMap C18) and eluted at a flow rate of 250 nL/min using a gradient of 4% to 11% B for 5 min; 11% to 32% B for 117 min; 32% to 50% B for 10 min; 50% to 97% B for 1 min, isocratic at 97% B for 17 min. Mobile phases A and B for the nano-LC were 0.1% FA in 2% ACN, and 0.1% FA in 88% ACN.

The data was collected (Xcalibur v 4.2.47, Thermo Fisher Scientific, USA) at the positive mode using data-dependent acquisition (DDA) with a 3 s cycle time. The MS1 spectra were acquired in the *m/z* range of 400–1500, with a resolution of 240 K (FWHM@*m/z* = 200). The maximum injection time for MS1 was 50 ms, automated gain control (AGC) target was 5E5. The dynamic exclusion was set to 60 s, and the mass tolerance was ±10 ppm. The precursor ions were filtered by quadruple with an isolation window of 1 Th and then fragmented by high energy collision dissociation (HCD) at a normalized collision energy of 30%. The MS/MS spectra were acquired under a resolution of 15 K (FWHM@*m/z* = 200) using orbitrap. MS/MS maximum injection time was 22 ms, and the AGC target was 5E4.

### Generation of protein distribution maps and further data processing using UHR-IonStar and the MAsP app

Protein identification and quantification were conducted using UHR-IonStar, a unique MS1-based quantitative proteomics pipeline, which achieves accurate and robust proteomic quantification in large cohorts, including low-abundance, regulatory proteins^15^. UHR-IonStar measures peptide MS1 precursor ions with ultra-high-resolution (UHR, FWHM = 240k@*m/z* = 200), and then precisely extract UHR-MS1 signals from the typically noisy backgrounds with extremely narrow, dynamically-defined *m/z* windows (e.g., 5 ppm) without losing signal intensity. This strategy effectively improves the selectivity and sensitivity for quantification of low-abundance proteins; along with an efficient chromatogram alignment approach, and a stringent post-feature quality control method, the UHR-IonStar showed accurate, precise quantification of large cohorts with low missing data and low false-positives^15,16^. Briefly, the LC-MS.raw files were converted to.mzXML format using ProteomicsTools and then the MS/MS spectra were searched against the Uniprot-SwissProt mouse database (16,961 entries, 2018) with the MS-GF + search engine (v 10089). A decoy database containing reverse sequences was concatenated to the forward database to allow the estimation of the false discovery rate (FDR) of the protein identification. The precursor ion mass tolerance was set to 20 ppm. The static modification was set to the carbamidomethylation of cysteine; the dynamic modification includes oxidation of methionine and acetylation of the N-terminal. Only fully tryptic peptides were considered. Each identified protein contains at least one unique peptide. The protein identification FDR was controlled on the entire dataset level at 1% using IDPicker (v 3.1.643.0). The quantitative feature annotation and further quantitative data processing were done by the UHR-IonStar package (v 1.5: https://github.com/JunQu-Lab/UHRIonStarApp). Briefly, MS1-peptide chromatographic peaks were aligned using the SIEVE (v 2.2.58) ChromAlign function to correct variations in chromatographic separation. The quantitative MS1 feature generation was conducted by a unique direct ion-current extraction (DICE) approach built in UHR-IonStar, which extracts the ultra-high-resolution MS1 peaks within precisely-defined, narrow *m/z* windows to enable sensitive and reliable quantification of all proteins including low-abundance proteins, as shown previously^15^. The quantitative features were annotated with peptide IDs via a precise matching^16^, and then quantitative values were aggregated from feature-level to peptide-level and then the protein-level.

To minimize the technical variations and achieve reproducible quantification, the total ion intensity (on the log2 scale) in each sample was normalized using the Eq. (1):

1 $${P}_{{ij}}\left({{{{{\rm{TIC}}}}}}\mbox{-}{{{{{\rm{normalized}}}}}}\right)={P}_{{ij}}-\,\left(\frac{1}{m}\mathop{\sum }\limits_{j=1}^{m}{P}_{{ij}}-\,\frac{1}{{mn}}\mathop{\sum }\limits_{i=1}^{n}\mathop{\sum }\limits_{j=1}^{m}{P}_{{ij}}\right)$$

where *P*_*ij*_ is the peptide abundance of *j*^*th*^ *(j* = *1, 2, …, m)* peptide in *i*^*th*^ *(i* = *1, 2, …, n)* sample after binary logarithmic transformation. For quantitative map generation, we further normalized the protein abundances against the abundances of these proteins quantified in the pooled reference samples (i.e., the QC samples) analyzed at the same time period as the micro-specimens. The users also have the option to use z-scores for map generation with the MAsP app, which allows a fair comparison/display of the spatial distribution of multiple proteins in the tissue slice.

The graphical user interface (GUI)-based MAsP app was developed to generate protein distribution maps as well as to perform further analysis of the maps. The app encompasses three primary functions (Supplementary Fig. 7): (1) generate customizable protein distribution maps based on the spatial coordinates and the protein abundances or z-scores, for specific proteins or all proteins in the dataset; (2) analyze protein distribution patterns from the thousands of generated protein distribution maps to identify proteins with non-random, regional distribution patterns using a previously-published approach^38^; discover protein maps with similar regional distribution patterns by the spectral clustering algorithm^19^. (3) among all MASP-generated maps, identify correlated distribution patterns between protein maps, or find protein maps that have correlated distribution patterns with that of a protein of interest, based on either Pearson correlation coefficient or cosine similarity. A manual with detailed information on the MAsP app can be found at https://github.com/JunQu-Lab/MAsP.

### Validation of the accuracy of the mapping technology

Three different approaches were used to validate the MASP pipeline. To examine the quantitative accuracy of the mapping strategy, non-endogenous peptides were spiked into the samples at specific locations to form a specific distribution pattern as shown in Fig. 3c. Three non-endogenous peptides (GPSVFPLAPSSK, LLINVGSR, LLIIGASTR) were spiked into the micro-specimens obtained from the designated location, at five different levels (0.5-, 0.7-, 1-, 1.3- and 2-fold at low fmol levels, respectively). These levels were designed to examine the ability of the method to discover relatively subtle differences among locations. The sequences of the peptides were concatenated into an artificial protein entry and added to the mouse Uniprot Swiss-Prot database. Based on the quantified protein abundance values, a distribution map of these peptides was then constructed and compared with the theoretical map based on the spiked-in ratios across the locations (Fig. 3c and Supplementary Fig. 10).

To corroborate the cerebral distribution of certain proteins with literature-reported patterns, a list of region-enriched proteins was identified from the previous proteomic studies^22,24^. The region-enriched expression patterns of these proteins were confirmed in the MASP-generated distribution maps. The functional annotation and pathway analysis of the quantified proteins were performed using the DAVID Functional Annotation (v 6.8)^39^ and KEGG Pathways (v 101.0)^40^. The distribution map of the first matched protein was shown in the figure when multiple corresponding genes are listed under one protein in the KEGG pathway map. Additional KEGG pathways and Gene Ontology functional annotation terms can be found in Supplementary Data 3, 4.

### Reporting summary

Further information on research design is available in the Nature Portfolio Reporting Summary linked to this article.

## Supplementary information

Supplementary Information

Reporting Summary

Description of Additional Supplementary Files

Supplementary Movie 1

Supplementary Data 1

Supplementary Data 2

Supplementary Data 3

Supplementary Data 4

## Data Availability

All data presented in this manuscript are available as supplementary data files. The LC-MS raw data generated in this study have been deposited in the ProteomeXchange Consortium via the PRIDE partner repository with the dataset identifier PXD037041 (MASP micro-specimen samples at different spatial locations). Uniprot-SwissProt mouse database (version downloaded on 07/13/2018, 16,961 entries). Source data are provided with this paper.

## Source data

Source Data

## Code availability

The code included in UHR-IonStar data processing is available at https://github.com/JunQu-Lab/UHRIonStarApp. The code included in MAsP app is available at https://github.com/JunQu-Lab/MAsP, and are archived on Zenodo (10.5281/zenodo.7320146)^41^.

## References

[1] Lu G (2020). Co-administered antibody improves penetration of antibody–dye conjugate into human cancers with implications for antibody–drug conjugates. Nat. Commun..

[2] Lee CM, Tannock IF (2010). The distribution of the therapeutic monoclonal antibodies cetuximab and trastuzumab within solid tumors. BMC Cancer.

[3] Besse, H. C., et al. Tumor drug distribution after local drug delivery by hyperthermia, In Vivo. *Cancers (Basel)* **11**, 1512 (2019). 10.3390/cancers11101512 PMC6826934 31600958

[4] Tan WCC (2020). Overview of multiplex immunohistochemistry/immunofluorescence techniques in the era of cancer immunotherapy. Cancer Commun..

[5] Uhlen M (2016). A proposal for validation of antibodies. Nat. Methods.

[6] Ryan DJ, Spraggins JM, Caprioli RM (2019). Protein identification strategies in MALDI imaging mass spectrometry: a brief review. Curr. Opin. Chem. Biol..

[7] Schulz S, Becker M, Groseclose MR, Schadt S, Hopf C (2019). Advanced MALDI mass spectrometry imaging in pharmaceutical research and drug development. Curr. Opin. Biotechnol..

[8] Zhu Y (2018). Nanodroplet processing platform for deep and quantitative proteome profiling of 10–100 mammalian cells. Nat. Commun..

[9] Budnik B, Levy E, Harmange G, Slavov N (2018). SCoPE-MS: mass spectrometry of single mammalian cells quantifies proteome heterogeneity during cell differentiation. Genome Biol..

[10] Zhu Y (2018). Proteomic analysis of single mammalian cells enabled by microfluidic nanodroplet sample preparation and ultrasensitive NanoLC-MS. Angew. Chem. Int. Ed. Engl..

[11] Kelly RT (2020). Single-cell proteomics: progress and prospects. Mol. Cell Proteom..

[12] Petyuk VA (2007). Spatial mapping of protein abundances in the mouse brain by voxelation integrated with high-throughput liquid chromatography-mass spectrometry. Genome Res..

[13] Piehowski PD (2020). Automated mass spectrometry imaging of over 2000 proteins from tissue sections at 100-μm spatial resolution. Nat. Commun..

[14] Mund A (2022). Deep Visual Proteomics defines single-cell identity and heterogeneity. Nat. Biotechnol..

[15] Wang X (2021). Ultra-high-resolution ionstar strategy enhancing accuracy and precision of MS1-based proteomics and an extensive comparison with state-of-the-art SWATH-MS in large-cohort quantification. Anal. Chem..

[16] Shen X (2018). IonStar enables high-precision, low-missing-data proteomics quantification in large biological cohorts. Proc. Natl Acad. Sci. USA.

[17] Shen S (2018). Surfactant cocktail-aided extraction/precipitation/on-pellet digestion strategy enables efficient and reproducible sample preparation for large-scale quantitative proteomics. Anal. Chem..

[18] Shen X (2017). An IonStar experimental strategy for MS1 ion current-based quantification using ultrahigh-field orbitrap: reproducible, in-depth, and accurate protein measurement in large cohorts. J. Proteome Res..

[19] Tung F, Wong A, Clausi DA (2010). Enabling scalable spectral clustering for image segmentation. Pattern Recognit..

[20] Xiao X (2018). Lymphotoxin β receptor-mediated NFκB signaling promotes glial lineage differentiation and inhibits neuronal lineage differentiation in mouse brain neural stem/progenitor cells. J. Neuroinflammation.

[21] Rozenblum GT, Kaufman T, Vitullo AD (2014). Myelin basic protein and a multiple sclerosis-related MBP-peptide bind to oligonucleotides. Mol. Ther. Nucleic Acids.

[22] Sharma K (2015). Cell type– and brain region–resolved mouse brain proteome. Nat. Neurosci..

[23] Morita K, Sasaki H, Fujimoto K, Furuse M, Tsukita S (1999). Claudin-11/OSP-based tight junctions of myelin sheaths in brain and Sertoli cells in testis. J. Cell Biol..

[24] Jung SY (2017). An anatomically resolved mouse brain proteome reveals parkinson disease-relevant pathways. Mol. Cell Proteom..

[25] Desai A, Mitchison TJ (1997). Microtubule polymerization dynamics. Annu. Rev. Cell Dev. Biol..

[26] Lavoie L, Levenson R, Martin-Vasallo P, Klip A (1997). The molar ratios of α and β subunits of the Na+−K+-ATPase differ in distinct subcellular membranes from rat skeletal muscle. Biochemistry.

[27] Gasiorowska, A. et al. The biology and pathobiology of glutamatergic, cholinergic, and dopaminergic signaling in the aging brain. *Front**Aging Neurosci*. **13** 654931 (2021). 10.3389/fnagi.2021.654931 PMC8315271 34326765

[28] Irvine GB, El-Agnaf OM, Shankar GM, Walsh DM (2008). Protein aggregation in the brain: the molecular basis for Alzheimer’s and Parkinson’s diseases. Mol. Med..

[29] Ross CA, Poirier MA (2004). Protein aggregation and neurodegenerative disease. Nat. Med..

[30] Lin L, Yee SW, Kim RB, Giacomini KM (2015). SLC transporters as therapeutic targets: emerging opportunities. Nat. Rev. Drug Discov..

[31] Daneman, R. & Prat, A. The blood–brain barrier. *Cold Spring Harb. Perspect. Biol*. **7** (2015). 10.1101/cshperspect.a020412 PMC4292164 25561720

[32] Pardridge WM (2012). Drug transport across the blood-brain barrier. J. Cereb. Blood Flow. Metab..

[33] Kadry H, Noorani B, Cucullo L (2020). A blood–brain barrier overview on structure, function, impairment, and biomarkers of integrity. Fluids Barriers CNS.

[34] Hughes CS (2019). Single-pot, solid-phase-enhanced sample preparation for proteomics experiments. Nat. Protoc..

[35] Li J (2020). TMTpro reagents: a set of isobaric labeling mass tags enables simultaneous proteome-wide measurements across 16 samples. Nat. Methods.

[36] Shen S (2021). Parallel, high-quality proteomic and targeted metabolomic quantification using laser capture microdissected tissues. Anal. Chem..

[37] An B (2020). Toward accurate and robust liquid chromatography–mass spectrometry-based quantification of antibody biotherapeutics in tissues. Anal. Chem..

[38] Fonville JM (2012). Robust data processing and normalization strategy for MALDI mass spectrometric imaging. Anal. Chem..

[39] Huang DW, Sherman BT, Lempicki RA (2009). Bioinformatics enrichment tools: paths toward the comprehensive functional analysis of large gene lists. Nucleic Acids Res..

[40] Kanehisa M, Sato Y, Kawashima M, Furumichi M, Tanabe M (2016). KEGG as a reference resource for gene and protein annotation. Nucleic Acids Res..

[41] Ma, M. et al. In-depth mapping of protein localizations in whole tissue by micro-scaffold assisted spatial proteomics (MASP). *Zenodo*10.5281/zenodo.7320146 (2022). 10.1038/s41467-022-35367-2 PMC9751300 36517484

