# Supplementary material for: In-depth mapping of protein localizations in whole tissue by micro-scaffold assisted spatial proteomics (MASP)
Source: Nat Commun. 2022 Dec 14;13:7736. doi: 10.1038/s41467-022-35367-2 (PMC9751300; doi:10.1038/s41467-022-35367-2)
Supplement: Supplementary file 1 — Supplementary Information [file 41467_2022_35367_MOESM1_ESM.pdf]

SUPPLEMENTARY INFORMATION

In-depth Mapping of Protein Localizations in Whole Tissue by Micro-scaffold Assisted Spatial Proteomics (MASP)

Min Ma[#1,2], Shihan Huo[#1], Ming Zhang[1,3], Shuo Qian[1,2], Xiaoyu Zhu[1], Jie Pu[1], Sailee Rasam[4], Chao Xue[5], Shichen Shen[1,3], Bo An[1,6], Jianmin Wang[2], Jun Qu*[1,2,3]

[1]Department of Pharmaceutical Sciences, SUNY at Buffalo, Buffalo, NY 14214;

[2]Roswell Park Comprehensive Cancer Center, Buffalo, NY 14203;

[3]New York State Center of Excellence in Bioinformatics & Life Sciences, Buffalo, NY 14203;

[4]Department of Biochemistry, Jacobs School of Medicine and Biomedical Sciences, SUNY at Buffalo, Buffalo, NY 14203;

[5]Department of Chemical and Biological Engineering, SUNY at Buffalo, Buffalo, NY 14214;

[6]Biotechnology, Huiyu (Seacross) Pharmaceuticals Ltd, Chengdu, China, 610219

*Author for correspondence: Tel.: +1 716 645 4821; Fax: +1 716 645 3693; junqu@buffalo.edu

# These authors contributed equally: Min Ma, Shihan Huo.

SUPPLEMENTARY NOTES

A. Development and optimization of the MASP strategy

1. Robust and uniform micro-compartmentalization of tissue slice while precisely preserving spatial information, a critical step to achieve accurate protein mapping on the whole-tissue level

1.1 The development of a 3D-printed micro-scaffold for precise and robust tissue compartmentalization

To achieve an accurate spatial mapping of proteins across the entire tissue slice, it is critical to precisely preserve the original spatial information when procuring the spatially specific micro-specimens. Moreover, the strategy should reproducibly recover micro-specimens of a uniform size, with high throughput and minimal cross-contamination. Traditional tissue micro-sampling strategies such as punch- or needle-biopsy do not meet these important requirements. Our initial attempts to obtain numerous spatial specimens from a tissue slice using needle biopsy for spatial tissue sampling showed that the process is very time-consuming and labor-intensive; more importantly, sequential needle sampling across different locations severely distorted the spatial information. To overcome these issues, we designed an arrays-style spatial micro-sampling device, referred to as a *micro-scaffold,* which contains precisely spaced micro-wells (shown in **Fig. 2a** and **Supplementary Fig. 1a**). The device acquires spatially-resolved micro-specimens with a one-strike cutting (*i.e., compartmentalization*) without distorting the spatial information, and each of the micro-specimens was evenly separated and sequestered inside the micro-wells of the device. In the example shown in **Fig. 2a**, the micro-scaffold contains 30 × 30 micro-wells, each with a cross-sectional dimension of 400 μm × 400 μm.

To minimize shift/distortion of the tissue slice during the compartmentalization process, each micro-well was designed with tapered edges. The narrower edges effectively and evenly immobilize the tissue slice upon the initial contact and further prevent horizontal motion of the tissue regions during the compartmentalization process, which precisely preserves spatial information (shown in the zoomed panel of **Fig. 2a** and **Supplementary Fig. 1a**). To fabricate the micro-scaffolds, we chose 3D printing for its flexibility in model design and quick turnover in testing and optimization. A number of state-of-the-art 3D-printing technologies were carefully evaluated. In this study, the ideal 3D-printing technique should carry an ultra-high printing resolution that is necessary to precisely construct the numerous micro-wells in the device, as well as a smooth inner surface to permit the complete and reproducible transfer of micro-specimens out of the wells after compartmentalization. Moreover, the fabricated device should carry excellent uniformity in all directions and the 3D-printing technique should be easily implemented in-house, enabling rapid production, testing, and optimization of designs. Several categories of 3D printing techniques were assessed, including fused deposition modeling (FDM), powder bed fusion (such as Selective Laser Sintering (SLS), Selective Laser Melting (SLM)), and photopolymerization (such as Stereolithography (SLA), Digital Light Processing (DLP))[1,2]. Our initial tests found

strategies such as FDM and SLS did not yield sufficient resolution or robustness for the fabrication of micro-scaffolds. By comparison, the photopolymerization-based techniques, which employ ultraviolet (UV) light to solidify the liquid photocurable polymer, afford the highest printing resolution with excellent precision. SLA and DLP are the two most popular types of techniques in this category, and examples include Low Force Stereolithography (LFS), Advanced-DLP, and Projection Micro Stereolithography (PμSL). After careful evaluation of these techniques, we selected the Advanced-DLP on desktop 3D printer (D4Kpro, EnvisionTEC, Germany), owing to its ultra-high printing resolution (~ 25 μm at the X and Y axes, and 1 μm at Z-axis), high printing speed and reasonable cost. As to the photopolymer, we chose HTM140v2, which is capable of high-resolution printing and meanwhile affords excellent strength for compartmentalization. The combination of the ultra-high resolution Advanced-DLP and the optimal photopolymer enabled the printing of the micro-scaffold with exceptional accuracy and precision (**Fig. 2a** and **Supplementary Fig. 1a**), as well as a smooth inner surface, thus laying a solid foundation for precise, robust spatial micro-compartmentalization of tissue slices.

1.2 The optimization of the supporting matrix for robust and complete tissue compartmentalization

In our pilot study, we found that to achieve complete separation of individual tissue micro-specimens, it is important to push the micro-scaffold downward till it cuts beyond the tissue layer into a supporting matrix. An ideal supporting matrix should have sufficient elasticity so that under pressurization it can protrude into the micro-wells to facilitate effective shearing of tissue against the narrow edges, and then achieving separation of individual tissue micro-specimens (**Supplementary Fig. 2**). The material should be strong enough to sustain the pressure and meanwhile be rupture-resistant, so that it can be intactly retracted from the micro-scaffold after the compartmentalization, without leaving pieces inside the micro-wells. Moreover, the material should not contain proteins (*i.e.,* causing contamination of the tissue proteome) or extractable polymers that can't be removed by the μ-SEPOD (discussed later) approach which could be detrimental to LC-MS analysis. Here we evaluated several candidate materials, including starch-based (*e.g.,* Play-Doh), PVC-based (*e.g.,* polymer clay), and silicon-based (*e.g.,* Polydimethylsiloxane-PDMS) modeling materials. Based on the results, we selected the PDMS because of its excellent strength and meanwhile optimal elasticity, and that no contamination proteins or detrimental polymers were introduced to the micro-specimens.

1.3 The development of a pressurization module for reproducible and uniform compartmentalization and micro-sampling

As discussed previously, a uniform and well-controlled pressurization across the entire tissue slice is an important prerequisite for robust and reproducible compartmentalization. To achieve this, we designed, fabricated, and optimized a 3D-printed pressurization-control module (**Fig. 2b** and **Supplementary Fig. 1b-c**), which includes a top and a bottom part that together encloses the stack of micro-scaffold, a tissue slice, and the supporting matrix (upper-to-lower order). The regulated

pressure is applied on the top part of the module, which transduces the pressure evenly to the micro-scaffold, facilitating uniform compartmentalization (**Supplementary Movie 1**). The design of the module was carefully optimized to ensure precise fitting, alignment, and leveling of components. The detailed parameters and photos of the products are shown in **Supplementary Fig. 1b-c.**

1.4 Optimization of the micro-compartmentalization procedure

In our pilot experiments, we observed that pre-freeze the tissue slice prior to the procedure is important to achieve reproducible compartmentalization. When pressing the micro-scaffold against a fresh brain tissue slice, the resulting micro-specimens are often of varied sizes, likely because of the variable stiffness and density among different anatomical regions[3]. To address this issue, we froze the tissue slice (-80 °C, ~ 30 min) immediately after slicing, which was found to have yielded micro-specimens with uniform sizes and high reproducibility. Compared to fresh tissue slices, pre-freezing the slice achieved much-improved reproducibility, robustness, and uniformity in the size of micro-specimens, as well as more identifiable proteins (**Supplementary Fig. 3**).

As to the compartmentalization procedure, the pressure and time for the two steps were optimized to maximize precision, reproducibility, and robustness, and the final conditions are shown in **Methods**. As shown in **Fig. 2d,** under the optimized conditions, the entire tissue slice was uniformly and completely compartmentalized without any left-over tissue while the spatial information was faithfully maintained.

1.5 Efficient and reproducible recovery of micro-specimens with 3D-printed piston arrays under a well-regulated environment inside a procurement chamber

The next critical step following compartmentalization is an efficient and reproducible collection of micro-specimens from the micro-wells. Pilot studies showed that this step must be performed right after micro-compartmentalization and be finished within a short time period (*e.g.,* <4 h), as the prolonged incubation of the specimens inside the micro-scaffold not only caused significant tissue dehydration which renders difficulty in recovering the micro-specimens, but also may result in protein degradation. To enable the efficient, high-throughput transfer of micro-specimens out of the scaffold, we devised a series of 3D-printed piston arrays (a total of 16) with breakable pistons to rapidly procure the tissue samples (**Supplementary Fig. 4a**). On each array, the pistons match one out of every four micro-wells in both horizontal and vertical directions. As shown in **Supplementary Fig. 4a**, the positions of the pistons are strategically staggered among the 16 arrays to cover all the micro-wells. All pistons were chamfered at 3.95 mm above the base to render them breakable, so that the upper part of the pistons carrying tissue specimens can be readily clipped off with a pair of lab forceps, which were then transferred into low-protein-binding tubes for subsequent sample preparation. With the piston arrays, we can rapidly collect >200 micro-specimens within 1 hour (**Supplementary Fig. 4b**). The tissues on the pistons were efficiently

extracted using μ-SEPOD (discussed below), and after centrifugation, the pistons were discarded along with the insoluble cellular components.

Furthermore, the above step must be performed under well-regulated conditions, including high humidity (~70-80%) to slow down tissue dehydration, low temperature (-5 to 0°C) to minimize protein degradation, and free of oxygen to eliminate protein oxidation. To maintain these conditions, we devised a tissue procurement chamber (**Supplementary Fig. 5**) equipped with all-angle LED lights to allow shadow-free operation, a controlled humidifier to provide the desired high humidity, and a dry ice container to provide low temperature and saturated CO_2 in the chamber. Compared to operation in the open air, procurement of micro-specimens in the chamber improved the success rate (*i.e.,* fully recovered specimens) from 50-60% to 100%.

2. Efficient/reproducible sample preparation and LC-MS analysis, robustly across all micro-specimens, another important prerequisite for reliable, quantitative mapping of tissue proteins

After compartmentalization, numerous micro-specimens were recovered. To realize reliable and accurate mapping of proteins, it is critical to achieve reproducible sample preparation (*i.e.,* protein extraction, sample cleanup, and digestion) with high and consistent efficiency across the many tiny micro-specimens (~100 μg tissue), as well as a sensitive and in-depth LC-MS analysis robustly across this large cohort of samples. To address this daunting challenge, we optimized a *μ*-SEPOD method that provides efficient and reproducible sample preparation of large sample cohorts with high, quantitative recovery of the micro-size samples. We have also adopted a trapping-nano-LC-MS system that extensively and robustly separates peptides and acquires high-quality, ultra-high-resolution-MS1 signals with excellent sensitivity consistently across large biological sample sets for reliable analysis of the large cohort of the micro-specimens.

2.1 The optimization of microscale surfactant cocktail-aided extraction/precipitation/on-pellet digestion (*μ*-SEPOD) for reproducible and robust sample preparation across the large cohort of micro-specimens

Previously we described a surfactant cocktail-aided extraction/precipitation/on-pellet digestion (SEPOD) method for the preparation of large sample cohorts with high efficacy, consistency, and reproducibility[4]. Briefly, the proteins were exhaustively extracted from the tissue by an optimized combination of a strong surfactant cocktail, and then a precipitation step was employed to remove non-protein matrix components and surfactants, which are detrimental to digestion and/or LC-MS analysis. The protein pellet was then digested with high efficiency and finally resulted in a clean sample for LC-MS analysis. The core of this approach is the utilization of a high-concentration cocktail of surfactants, which provided three salient benefits, including near-complete protein recovery from tissues, extensive cleanup of the samples by removing matrix components with the surfactants, and rapid and highly efficient digestion owing to the “dual denaturation” by both the surfactants and precipitation[4]. Compared to other popular methods, the SEPOD provided markedly

higher and more reproducible recoveries of proteins and peptides from tissues, especially the membrane proteins[4].

In this study, we adapted the SEPOD approach to prepare the micro-size tissues with high and consistent efficiency across the large cohort of spatial micro-specimens. Several key parameters were rigorously optimized. Firstly, we assessed the use of 20, 30, and 50 μL surfactant cocktail buffer per sample, in terms of the extraction efficiency, reproducibility, and the number of quantifiable proteins. While using very low volumes of buffer caused incomplete extraction and significant variation, the use of volumes that is too large (*i.e.,* > 50 μL for 10 μg protein) would result in low protein concentrations (*i.e.,* < 0.2 mg/mL) which compromises the efficiency of SEPOD[4]. In our evaluations, it was observed that using 30 μL buffer (~1:300 tissue: buffer, w/v) resulted in the highest number of quantifiable proteins with excellent extraction efficiency and reproducibility (**Supplementary Fig. 6a**), and therefore this volume was selected. Secondly, to achieve a high and reproducible protein recovery, it is critical to thoroughly disrupt the cellular compartments to ensure a thorough protein recovery. Considering the tiny sizes of the micro-specimens, sonication is the only viable choice. Here we compared two sonication approaches: sonication via water bath for 15 min or using a high-power probe sonication for 5s per cycle, three cycles per sample, as established previously[4]. It was found sonication markedly improved protein extraction efficiency. Though there was no significant difference in protein yields between the two sonication strategies (**Supplementary Fig. 6b**), we selected water bath sonication because of the better reproducibility, as well as the easier operation with higher throughput. Moreover, the sonication step also effectively sheared large-molecule-weight nuclear acids into small fragments[4], which are then removed by the precipitation step.

Finally, protein loss owing to adsorption to the sample tubes is an important concern for the preparation of micro-size tissue samples[5], which must be evaluated. We found no perceivable protein loss during the extract step since the strong surfactant cocktail buffer prevented protein adsorption. Nonetheless, a substantial loss of peptides was observed during the surfactant-free digestion procedure when using a standard Eppendorf tube. We further discovered that the use of low-protein-binding tubes greatly alleviated this problem, which resulted in higher peptide recovery and more quantified proteins, as shown in **Supplementary Fig. 6c**.

Based on the above optimization results, an optimal μ-SEPOD protocol was developed, and the details are shown in **Methods.**

2.2 A sensitive and reproducible trapping nano-LC high-resolution MS system for high quantitative quality when analyzing large cohort samples

The other key component to assure the quantitative accuracy of MASP is a protocol for highly sensitive liquid chromatography-mass spectrometry (LC-MS) analysis, with excellent robustness and reproducibility across the large cohort of micro-specimens. Towards this end, we adopted a trapping nano-LC setup that consists of two synchronized LC systems: one micro-flow system utilizing a large inner diameter (I.D.) trapping column, and a nano-flow system using a 65 cm-long analytical column packed with small particles, which have achieved high-resolution

chromatographic separation with excellent analytical reproducibility among a large number of samples, as shown in the previous publications[6-8]. The large-I.D. trapping column is an important component in this system, as it enables highly robust, reproducible, and sensitive analysis of large cohorts. Specifically, *i)* selective trapping/delivery via the trapping column prevented hydrophilic/hydrophobic matrix components from entering the LC-MS, affording excellent robustness for analysis of many samples[7,8]; *ii)* the large-I.D. trapping column provided homogeneously mixed mobile phase to the nano-column, providing highly reproducible separation[6]; *iii)* the large-I.D. trapping column drastically increased the quantitative loading capacity of the system, and thereby substantially improved the signal-to-noise ratio (S/N) for low-abundance peptides that often represent proteins with critical biological functions[7].

Ultra-high-resolution (120K-240K FWHM@*m/z*=200) MS1 (UHR-MS1) detection was employed to attain highly selective and sensitive quantification by IonStar, as demonstrated previously[9]. For MS2 fragmentation, HCD/OT rather than HCD/IT mode was employed owing to its low false-positives, which is tremendously beneficial for large-cohort analysis[7].

This unique LC-MS strategy has enabled sensitive, comprehensive and reliable quantification of the many spatially resolved micro-specimens that were procured in this project. Moreover, we observed exceptional reproducibility and robustness across the analysis of all the micro-specimens, which have laid another solid foundation for reliable quantitative mapping by MASP.

3. Generation of protein distribution maps based on the accurate protein quantification by UHR-IonStar and the development of an R-based spatial mapping app, MAsP, with a graphical user interface (GUI)

3.1 Sensitive and accurate quantification of the proteins among the micro-specimens by the UHR-IonStar data processing pipeline

To achieve high-quality quantitative mapping, another important prerequisite is a data processing pipeline capable of sensitive, accurate and reproducible quantification of the many micro-specimens. Furthermore, a large-cohort analysis is often susceptible to problems such as high missing data and elevated false-positives[10,11]. Towards this end, the UHR-IonStar data processing approach, which achieves exceptional sensitivity and data quality for proteomics quantification in large cohorts, was employed[8,9]. UHR-IonStar measures peptide MS1 precursor ions with ultra-high-resolution (UHR, 240K FWHM@*m/z*=200), and then uses a novel approach that is to precisely extract UHR-MS1 signals from the typically noisy backgrounds with extremely narrow, dynamically-defined *m/z* windows (*e.g.,* 5 ppm) without losing signal intensity. This strategy effectively improved the selectivity and sensitivity for the quantification of low-abundance proteins. In addition, by employing an efficient chromatogram alignment approach, and a stringent post-feature quality control method, UHR-IonStar showed accurate, precise quantification of large cohorts with low missing data and low false-positives[8,9]. In this study, the UHR-IonStar method achieved high quantitative precision (median intra-group CV% for protein abundance values among QC replicates was 9.4%) and excellent reproducibility (Pearson correlation r=0.964-0.983 among sample preparation replicates), as shown in **Fig. 3a-b and Supplementary Fig. 9**.

Additionally, 5019 proteins were quantified (**Supplementary Data 1**), among which 98.2% (4929 proteins) were quantified in at least 95% of all spatial samples, 91.2 % (4577 proteins) were quantified in all regions of the brain slice (**Supplementary Data 2**); indicating the excellent capacity of UHR-IonStar for reproducible measurement in large cohorts.

3.2 The development of a MAsP app for the generation and processing of protein distribution maps

To facilitate rapid and versatile generation and analysis of protein distribution maps in tissues, we developed an R Shiny-based MAsP app (v1.0, https://github.com/JunQu-Lab/MAsP)(**Supplementary Fig. 7**). The basic function of this graphical user interface (GUI) app is to generate customizable protein distribution maps based on the spatial coordinates and the protein abundance or the z-score in each micro-specimen. The maps can be generated for either specific proteins or all proteins in the dataset. Parameters such as map resolution, colors, range of abundance/z-score values, and background transparency, can be customized by the users.

Furthermore, the MAsP app can discover protein distribution patterns among the thousands of protein distribution maps generated. To identify proteins with non-random, region-specific distribution patterns, the MAsP app utilizes a published approach that evaluates abundance distribution on maps using the parameter of the percentage of variance explained (VE) in the first singular value after factorization by a singular value decomposition (SVD) made for each protein distribution map[12]. For example, if values of abundance or z-scores across the whole tissue were randomly distributed, the VE by the first singular value would be very low. An SVD filtering with the user-defined cutoff of the VE threshold can be used to remove protein maps with random regional distribution patterns. Furthermore, the remaining maps with similar regional distribution patterns can be grouped by a spectral clustering algorithm, which is a density-based clustering algorithm designed for image processing[13].

Finally, proteins with correlated distribution patterns could imply co-localization of these proteins, which may provide highly valuable information on spatially organized biological processes. Here we devised a module to identify protein maps that have correlated distribution patterns with that of a protein of interest, among all MASP-generated maps in the dataset, based on either Pearson correlation coefficient or cosine similarity. A detailed manual about the MAsP app can be found at: https://github.com/JunQu-Lab/mAsP.

B. The MASP strategy was validated and showed high accuracy in quantitative mapping

We validated the accuracy of protein mapping by MASP in three different ways.

First, low, strategically varied levels of non-endogenous peptides were spiked into the micro-specimens of different locations so that a designed pattern (**Fig. 3c, theoretical pattern**) would show up in the maps of these peptides if MASP achieved accurate mapping. In brief, we spiked three non-endogenous peptides (GPSVFPLAPSSK, LLINVGSR and LLIIGASTR) at five different levels (0.5-, 0.7-, 1-, 1.3- and 2-fold at low fmol levels, respectively) into the micro-specimens at the designated locations (**Supplementary Fig. 10**). These levels were designed to

examine the ability of MASP to identify relatively subtle differences in protein abundances among locations. The sequences of the peptides were concatenated into an artificial protein entry, which was then integrated into the mouse proteome database, and quantified along with other proteins in the database. Based on the quantified abundance values, a distribution map of these peptides was constructed and compared with the theoretical distribution map calculated based on the spiked-in ratios across the locations (**Fig. 3c, MASP quantified pattern**). The result showed that MASP faithfully produced the expected patterns with excellent accuracy (mean error% = 12.9%).

Second, we further compared MASP-generated maps of certain proteins against their previously known distribution patterns, such as some literature-reported cell type markers and region-enriched markers. For example, the whole brain distribution of a well-known marker for oligodendrocytes, myelin basic protein (*Mbp*), has been well-documented[14-16]. Here we found highly correlated patterns between the MASP-generated map of *Mbp* and its previously-reported distributions[14]. We further compared the distribution of *Mbp* with four other oligodendrocytes markers: myelin oligodendrocyte glycoprotein (*Mog*), oligodendrocyte-specific protein (*Osp*), and 2',3'-cyclic-nucleotide 3'-phosphodiesterase (*Cnp*), and myelin proteolipid protein (*Plp*). As shown in **Fig. 3d**, the MASP-generated maps of these markers showed high similarity with the map of *Mbp* (Pearson r values of 0.95, 0.78, 0.99, and 0.97, respectively), suggesting reliable mapping by MASP. In addition, we also surveyed the maps of some protein markers that are known to be enriched in various brain anatomic regions[17,18]. MASP correctly recapitulated the expected protein distributions in these anatomical regions such as the cortex (*Cacng3, Slc30a3, Synpo*) and hypothalamus (*Ahi1, Baiap3, Scg2*), with high correlations (r= 0.81-0.95, **Fig. 3d**). More examples of regionally enriched proteins are shown in **Supplementary Fig. 11,** which include hippocampus-enriched proteins (*e.g., Acdy9*), cortex-enriched proteins (*e.g., Ansk1b*), striatum-enriched proteins (*e.g., Ppp1r1b*), as well as hypothalamus-enriched proteins (*e.g., Scg5*), where the MASP-generated protein distribution maps showed patterns consistent with literature[17,18].

Third, we tested the quantitative accuracy of MASP by correlating the spatial distributions of the two distinct proteins that form the same heterodimeric protein complex. In this study, only heterodimeric protein complexes that meet the following two criteria were chosen to validate the MASP technique: *i)* the two proteins in the heterodimer must have distinct, non-overlapping sequences, otherwise the impact of shared peptides might cause overestimation of mapping accuracy; and *ii)* the majority of the two proteins should exist in the heterodimer form so that these two proteins are co-located. Here, two such heterodimers are evaluated: Tubulin α-β heterodimer[19] and Na,K-ATPase α-β heterodimer[20,21]. If the MASP method could accurately recapitulate the spatial distribution of proteins, the maps of the two components of a heterodimer that are independently acquired by MASP, should show highly similar distribution patterns. Indeed, highly correlated patterns of the two components were observed for the two above-mentioned heterodimers (r= 0.93, and 0.95, respectively, **Fig. 3e**), which validates the accuracy of mapping by MASP.

SUPPLEMENTARY FIGURES

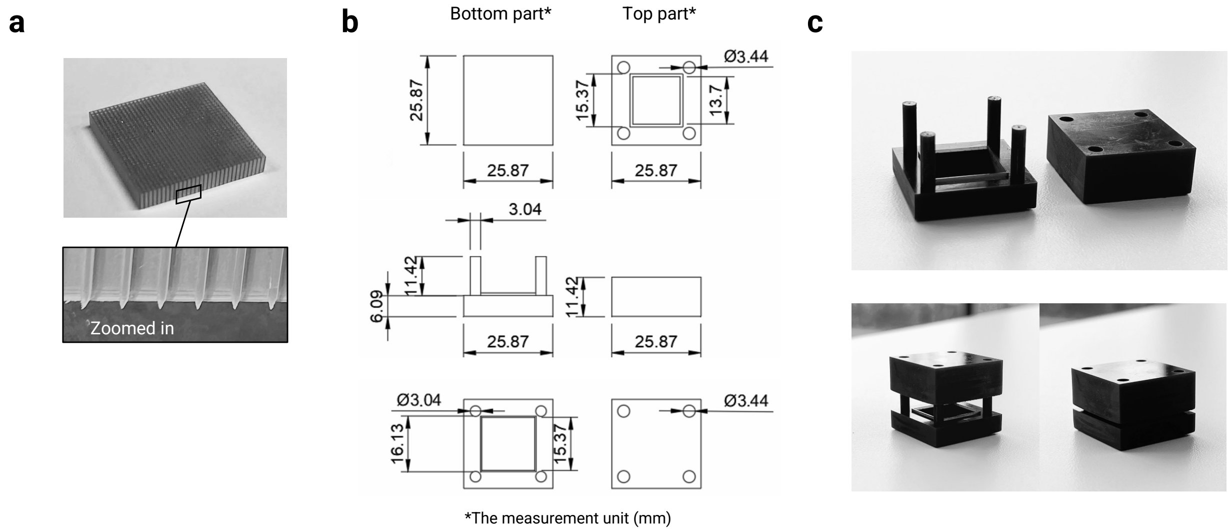

Fig. S1 | The main 3D printed devices involved in the spatially-resolved micro-compartmentalization. a, The photos of a 3D-printed micro-scaffold with sharp edges for precise and robust tissue compartmentalization. **b,** The design and measurements (in mm) of the pressurization module, which enables uniform and well-controlled pressurization during the compartmentalization procedure. **c,** The photo of the 3D-printed pressurization module.

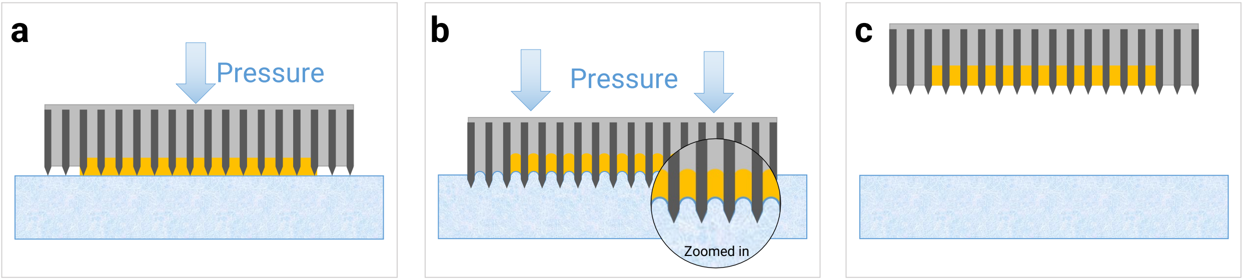

Fig. S2 | The rationale for that an optimal supporting matrix with proper elasticity and strength is important to enable complete tissue compartmentalization with segregated micro-specimens. a, The initial pressure is applied to the assembled micro-scaffold (gray), tissue slice (yellow), and the supporting matrix (blue), which prevents tissue motion. **b,** The supporting matrix protrudes into the micro-wells under the increased pressure, cutting off the tissue around the microwell. **c,** The supporting matrix remains intact after the retraction of the micro-scaffold, while the micro-specimens were individually sequestered in each micro-well.

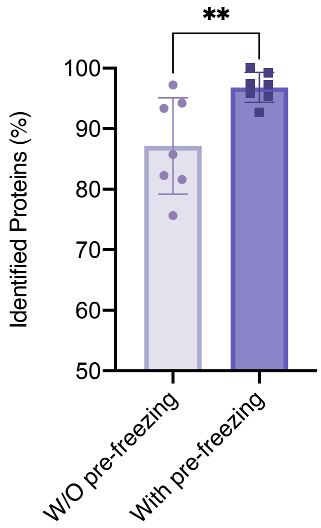

Fig. S3 | The identified proteins in the tissue slices with or without pre-freezing. (Student *t* test, 2-sided, t=3.076, df=12, p=0.0096, *: $p < 0.05$, **: $p < 0.01$, N=7 per group). Data are presented as mean±SD. df, degree of freedom. W/O pre-freezing: light purple. With pre-freezing: dark purple. Source data are provided as a Source Data file.

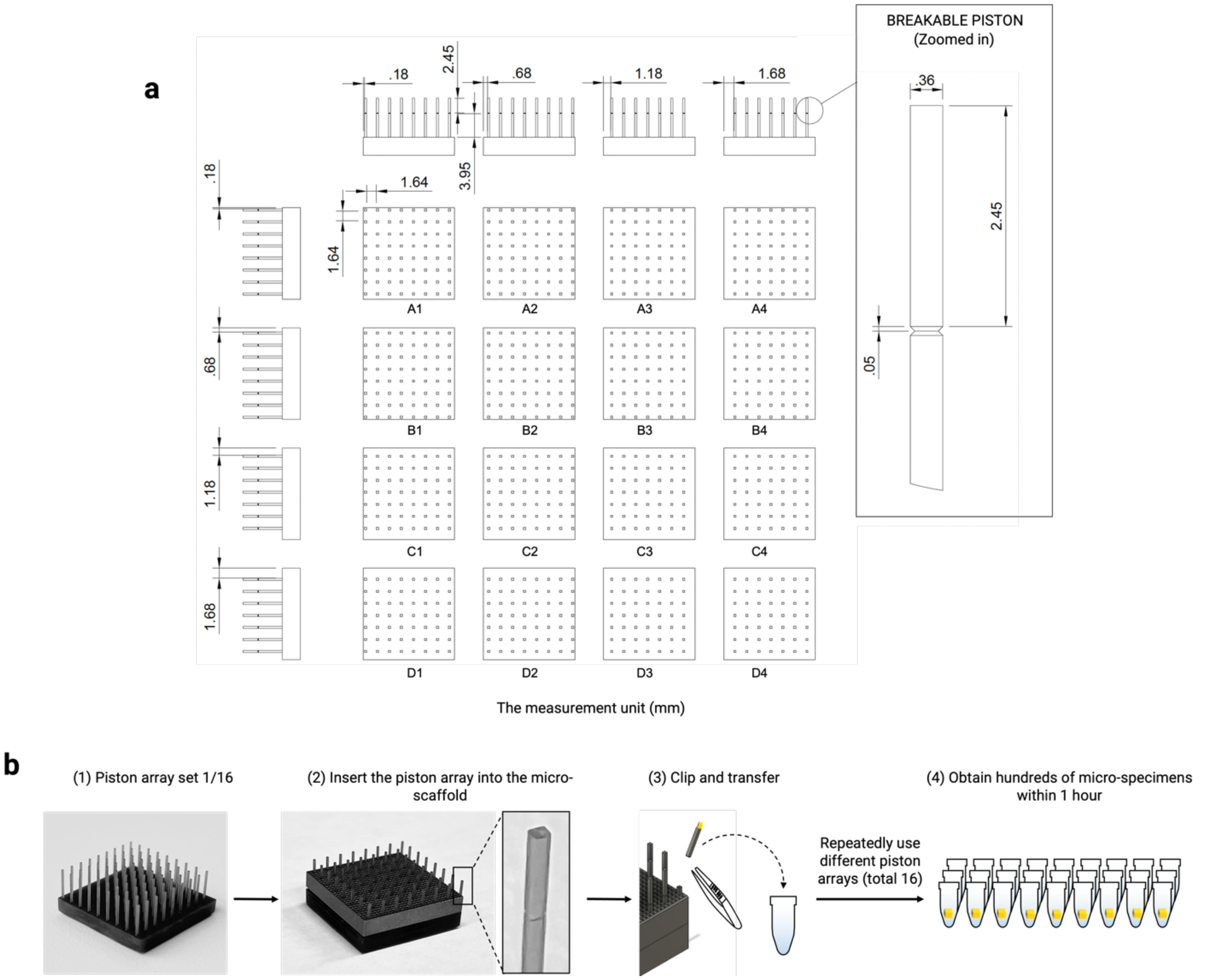

Fig. S4 | The 3D-printed piston arrays for efficient and reproducible recovery of micro-specimens. a, The design and measurements (in mm) of a series of staggered piston arrays, each matching 1 out every 4 micro-wells in both horizontal and vertical directions; **b,** The photos of the 3D-printed piston arrays and the scheme of the efficient procurement of micro-specimens. (1) The photo of a representative 3D-printed piston array with breakable pistons. (2) The photo of the micro-scaffold and the inserted piston array with breakable pistons showed in the zoomed-in window. (3) The clip and transfer of the piston, which carries the micro-specimen, into sample tubes. (4) Using the set of 16 piston arrays, each with staggered positions, rapid collection of all micro-specimens in the micro-scaffold can be achieved within 1 hour.

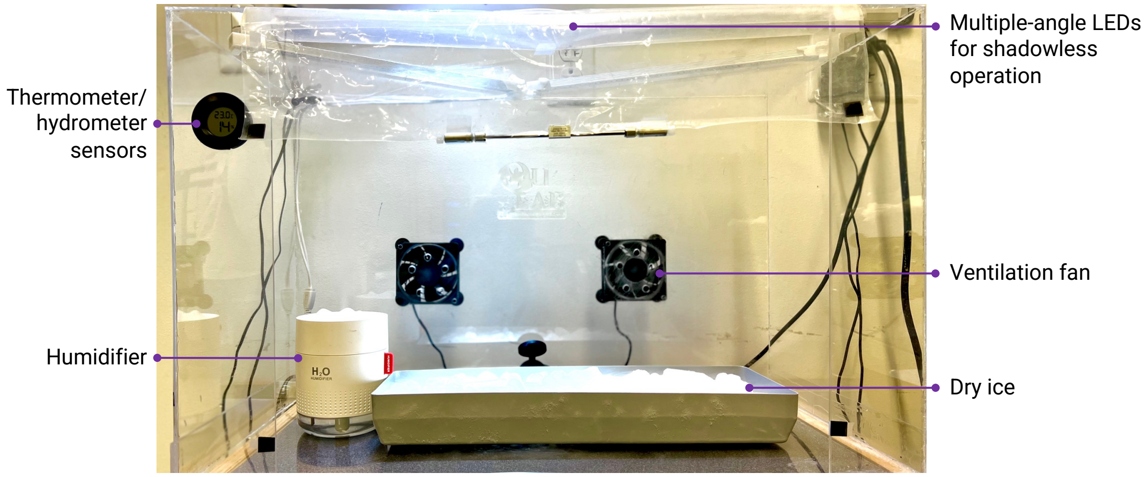

Fig. S5 | The procurement chamber with tightly regulated conditions for efficient and reproducible recovery of the micro-specimens. The chamber is equipped with all-angle LED lights to allow shadow-free operation, a controlled humidifier to provide the desired high humidity, and a dry ice container to provide low temperature and saturated CO_2 in the chamber.

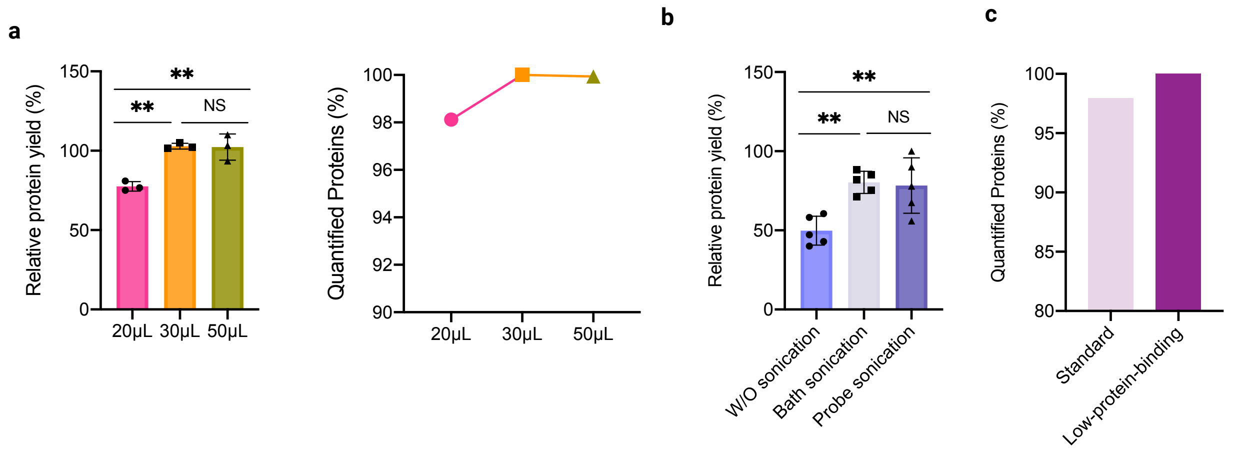

Fig. S6 | The optimization of some key parameters of the μ-SEPOD protocol, which permits the reproducible and robust sample preparation across the large cohort of micro-specimens. **a,** The protein yield (left) and quantified proteins (right) of micro-specimens extracted by 20μL (pink), 30μL (orange), or 50μL (olive) surfactant cocktail buffer. One-way ANOVA test: F (DFn, DFd)=23.21(2,6), *p*=0.0015, and Holm-Sidak's multiple comparisons test: 20μL vs. 30μL: p=0.0030, 20μL vs. 50μL: *p*=0.0030, 30μL vs. 50μL: *p*=0.8979. (*: $p < 0.05$, **: $p < 0.01$, NS: $p \geq 0.05$, N=3 per group). Data are presented as mean±SD. **b,** The protein concentration of micro-specimens without sonication (light purple) or with sonication by a water-bath sonicator (gray), or probe sonicator (dark purple). One-way ANOVA test: F (DFn, DFd)=9.947(2,12), *p*=0.0028, and Holm-Sidak's multiple comparisons test: W/O sonication vs. Bath sonication: *p*=0.0054, W/O sonication vs. Probe sonication: *p*=0.0058, Bath sonication vs. Probe sonication: *p*=0.7966. (*: $p < 0.05$, **: $p < 0.01$, NS: $p \geq 0.05$, N=5 per group). Data are presented as mean±SD. **c,** The quantified proteins from the micro-specimens using standard (light plum) or low-protein-binding Eppendorf tubes (dark plum). DFn, degrees of freedom in the numerator. DFd, degrees of freedom in the denominator. Source data are provided as a Source Data file.

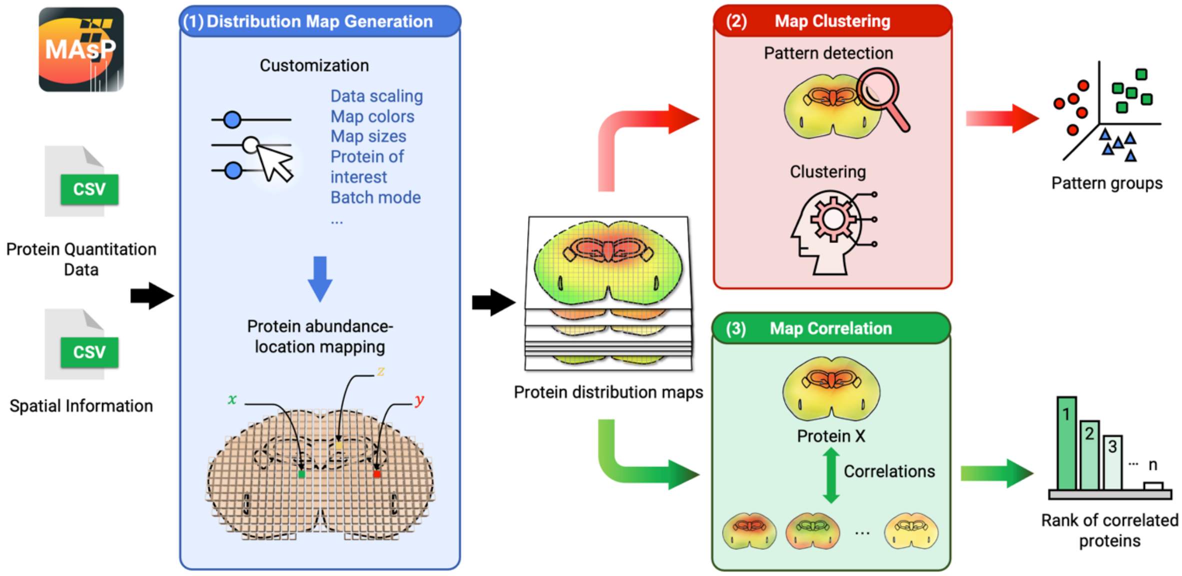

Fig. S7 | Schematic illustration of the data processing functions in MAsP app. The MAsP app encompasses three primary functions: (1) generate customizable protein distribution maps based on the spatial coordinates and the protein abundances or z-scores, either for specific proteins or all proteins in the dataset; (2) analyze protein distribution patterns from the thousands of generated protein distribution maps to identify proteins with non-random, regional distribution patterns; discover protein maps with similar regional distribution patterns by the spectral clustering algorithm; (3) among all MASP-generated maps, identify correlated distribution patterns between protein maps, or find protein maps that have correlated distribution patterns with that of a protein of interest, based on either Pearson correlation coefficient or cosine similarity.

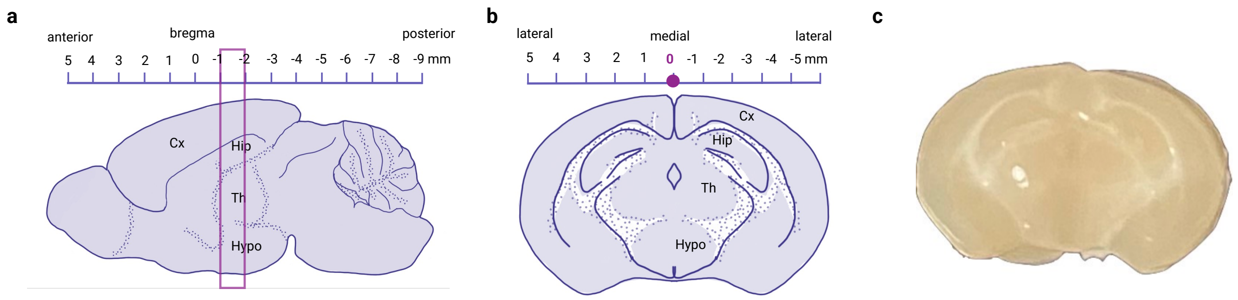

Fig. S8 | The anatomical position of the brain slice used in this study. a, The sagittal view of the brain slice which includes the main anatomical regions such as the cortex (Cx), hippocampus (Hip), thalamus (Th), and hypothalamus (Hypo). The 1-mm brain coronal slice was acquired at the anatomical coordinates of -1mm to -2mm posterior to bregma, denoted in the purple frame. **b,** The coronal view of the brain slice, and the main anatomical regions. **c,** the photo of a representative mouse brain coronal slice.

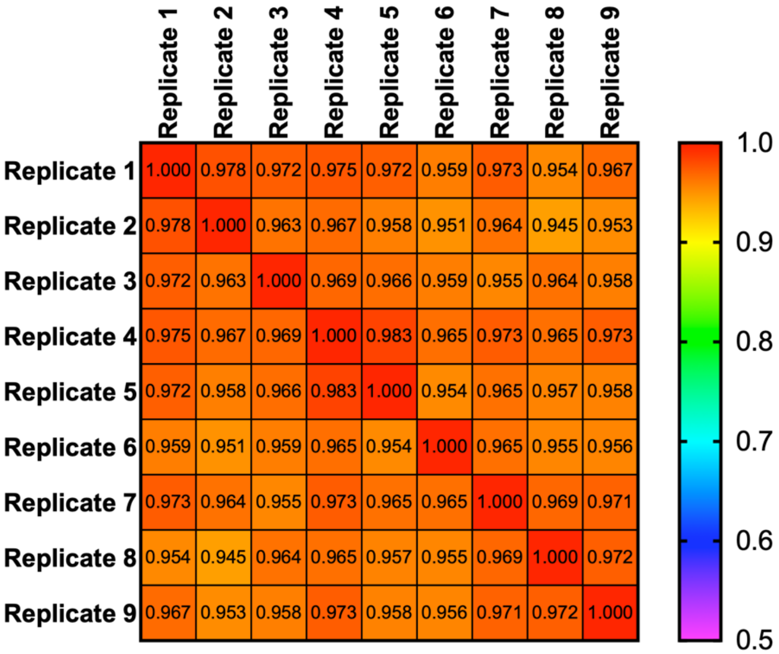

	Replicate 1	Replicate 2	Replicate 3	Replicate 4	Replicate 5	Replicate 6	Replicate 7	Replicate 8	Replicate 9
Replicate 1	1.000	0.978	0.972	0.975	0.972	0.959	0.973	0.954	0.967
Replicate 2	0.978	1.000	0.963	0.967	0.958	0.951	0.964	0.945	0.953
Replicate 3	0.972	0.963	1.000	0.969	0.966	0.959	0.955	0.964	0.958
Replicate 4	0.975	0.967	0.969	1.000	0.983	0.965	0.973	0.965	0.973
Replicate 5	0.972	0.958	0.966	0.983	1.000	0.954	0.965	0.957	0.958
Replicate 6	0.959	0.951	0.959	0.965	0.954	1.000	0.965	0.955	0.956
Replicate 7	0.973	0.964	0.955	0.973	0.965	0.965	1.000	0.969	0.971
Replicate 8	0.954	0.945	0.964	0.965	0.957	0.955	0.969	1.000	0.972
Replicate 9	0.967	0.953	0.958	0.973	0.958	0.956	0.971	0.972	1.000

Fig. S9 | Correlation between all sample preparation replicates (N=9) of QC samples, analyzed between every 20 micro-specimens runs. Source data are provided as a Source Data file.

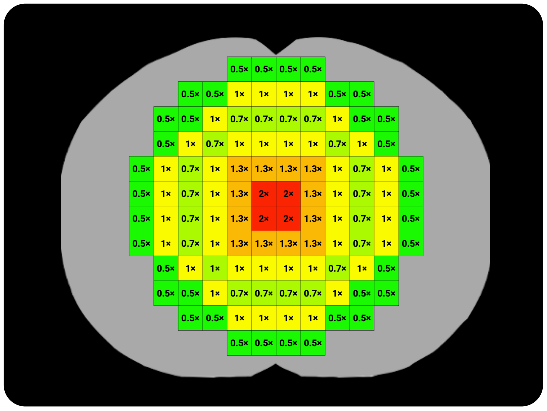

Fig. S10 | The designed locations and levels of the spiked-in non-endogenous peptides for the validation of the quantitative accuracy of MASP. The numbers denote the folds of spiked levels.

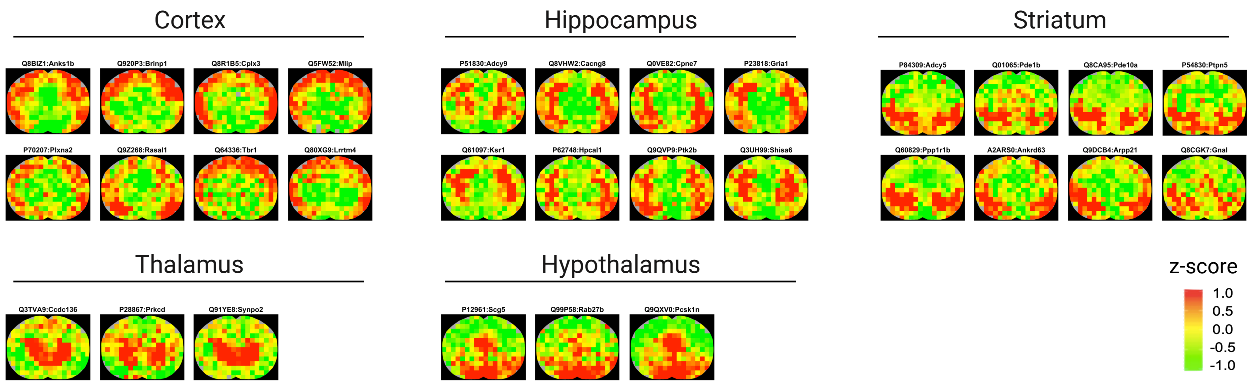

Fig. S11 | Additional results for validation of the mapping reliability by MAPS. The maps of more region-enriched proteins that are highly expressed in certain brain regions, which correlated well with the literature. The z-score color scale is from -1.0 (green) to 1.0 (red).

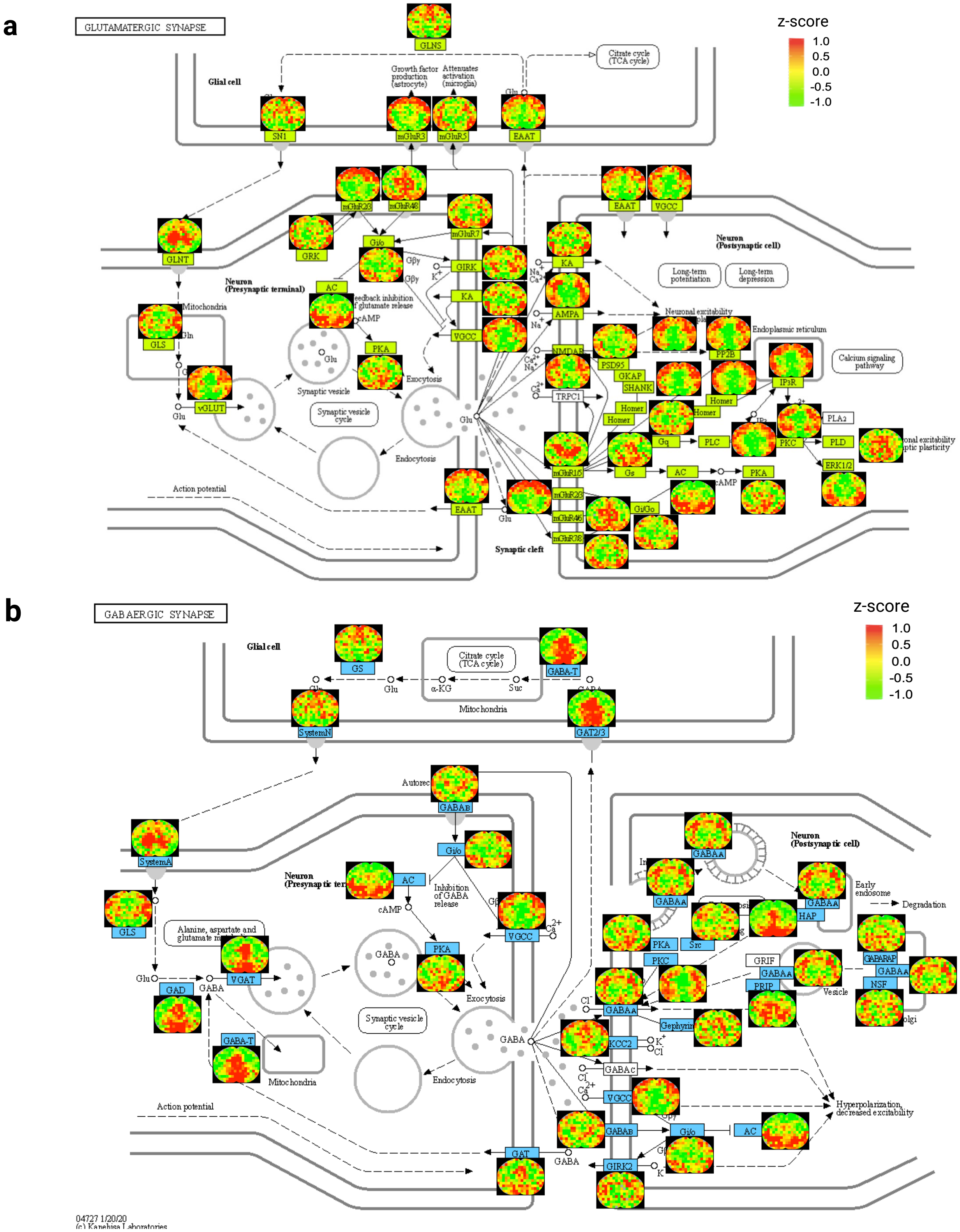
a
GLUTAMATERGIC SYNAPSE
z-score
1.0
0.5
0.0
-0.5
-1.0
Glial cell
GLNS
Citrate cycle (TCA cycle)
Growth factor production (astrocyte)
Attenuates activation (microglia)
SN1
mGluR3
mGluR5
EAAT
GLNT
GRK
Gi/o
GIRK
KA
VGCC
AC
PKA
Neuron (Presynaptic terminal)
Mitochondria
GLS
vGLUT
Synaptic vesicle
Synaptic vesicle cycle
Exocytosis
Endocytosis
Action potential
Synaptic cleft
Neuron (Postsynaptic cell)
Long-term potentiation
Long-term depression
AMPA
NMDAR
PSD95
GKAP
SHANK
Homer
TRPC1
Endoplasmic reticulum
PP2B
IP3R
Calcium signaling pathway
PLA2
PKC
PLD
ERK1/2
Gq
PLC
Gs
Gi/Go
b
GABAERGIC SYNAPSE
z-score
1.0
0.5
0.0
-0.5
-1.0
Glial cell
GS
Citrate cycle (TCA cycle)
GABA-T
Mitochondria
SystemN
GAT2/3
GABAB
SystemA
Gi/o
Neuron (Presynaptic terminal)
AC
cAMP
Inhibition of GABA release
GLS
Alanine, aspartate and glutamate metabolism
GAD
VGAT
PKA
VGCC
Synaptic vesicle cycle
Exocytosis
Endocytosis
Action potential
Neuron (Postsynaptic cell)
GABAA
Early endosome
Degradation
HAP
Src
PKC
GRIF
PRIP
GABARAP
NSF
Vesicle
Gephyrin
KCC2
GABAC
GIRK2
GAT
Hyperpolarization, decreased excitability
04727 1/20/20
(c) Kanehisa Laboratories

c

d

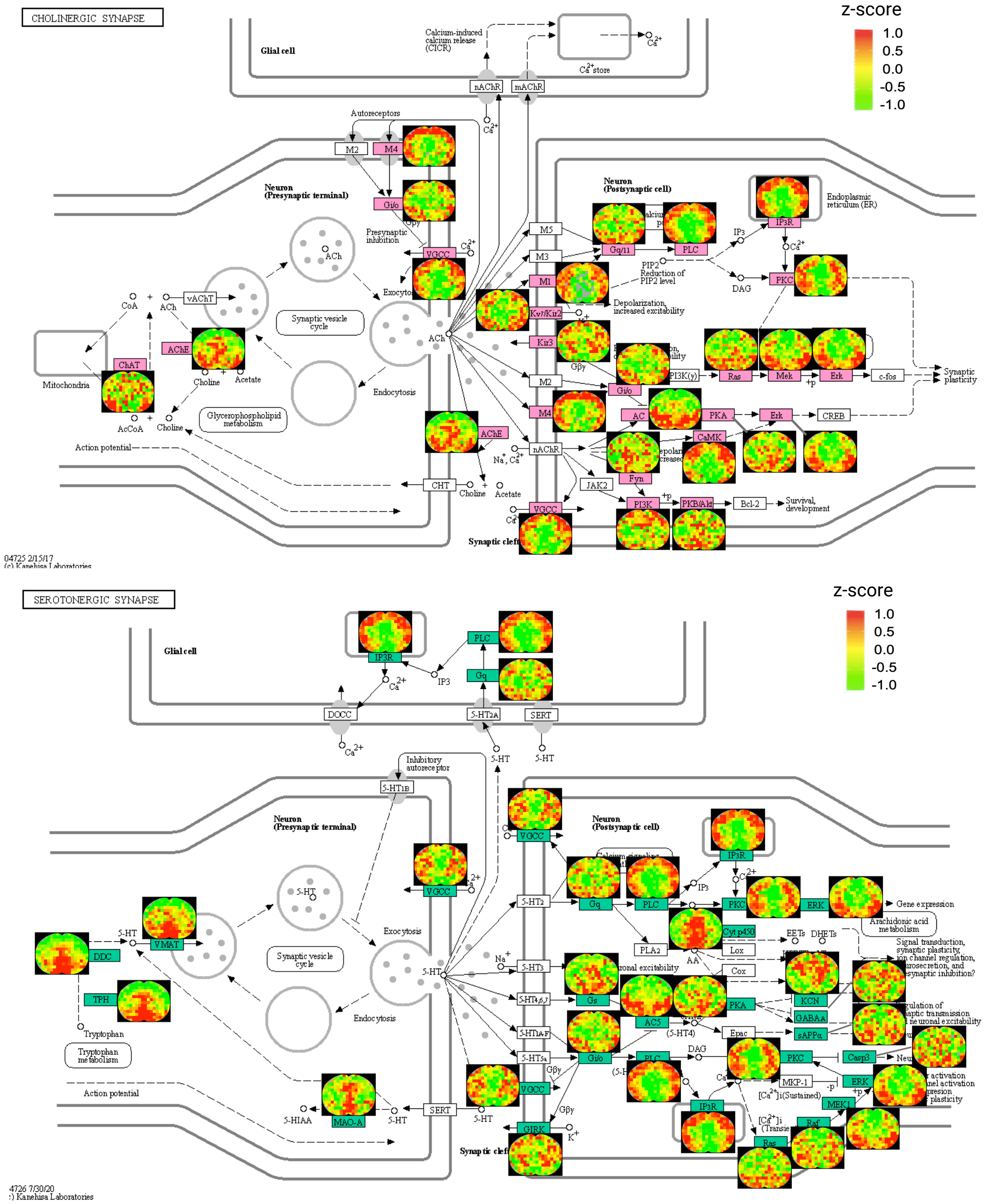

Fig. S12 | Cerebral distribution maps of the key proteins involved in synaptic pathways that are important for neurodegenerative diseases, cognitive function and aging. a, Glutamatergic synapse pathway, **b,** GABAergic synapse pathway, **c,** Cholinergic synapse pathway, and **d,** Serotonergic synapse pathway. The z-score color scale for **a-d** is from -1.0 (green) to 1.0 (red). The lime, blue, pink, and turquoise boxes on the KEGG pathway plots in **a**, **b**, **c**, and **d**, respectively, denote the proteins quantified by MASP.

References

1. Ngo, T. D., Kashani, A., Imbalzano, G., Nguyen, K. T. Q. & Hui, D. Additive manufacturing (3D printing): A review of materials, methods, applications and challenges. *Compos. B. Eng.* **143**, 172-196 (2018).
2. Bagheri, A. & Jin, J. Photopolymerization in 3D Printing. *ACS Appl. Polym. Mater.* **1**, 593-611 (2019).
3. Budday, S. *et al.* Towards microstructure-informed material models for human brain tissue. *Acta Biomater* **104**, 53-65 (2020).
4. Shen, S. *et al.* Surfactant Cocktail-Aided Extraction/Precipitation/On-Pellet Digestion Strategy Enables Efficient and Reproducible Sample Preparation for Large-Scale Quantitative Proteomics. *Anal. Chem.* **90**, 10350-10359 (2018).
5. Maes, K., Smolders, I., Michotte, Y. & Van Eeckhaut, A. Strategies to reduce aspecific adsorption of peptides and proteins in liquid chromatography–mass spectrometry based bioanalyses: An overview. *J. Chromatogr. A* **1358**, 1-13 (2014).
6. Nouri-Nigjeh, E. *et al.* Highly Multiplexed and Reproducible Ion-Current-Based Strategy for Large-Scale Quantitative Proteomics and the Application to Protein Expression Dynamics Induced by Methylprednisolone in 60 Rats. *Anal. Chem.* **86**, 8149-8157 (2014).
7. Shen, X. *et al.* An IonStar Experimental Strategy for MS1 Ion Current-Based Quantification Using Ultrahigh-Field Orbitrap: Reproducible, In-Depth, and Accurate Protein Measurement in Large Cohorts. *J Proteome Res* **16**, 2445-2456 (2017).
8. Shen, X. *et al.* IonStar enables high-precision, low-missing-data proteomics quantification in large biological cohorts. *Proc. Natl. Acad. Sci. USA* **115**, E4767 (2018).
9. Wang, X. *et al.* Ultra-High-Resolution IonStar Strategy Enhancing Accuracy and Precision of MS1-Based Proteomics and an Extensive Comparison with State-of-the-Art SWATH-MS in Large-Cohort Quantification. *Anal. Chem.* **93**, 4884-4893 (2021).
10. Domon, B. & Aebersold, R. Options and considerations when selecting a quantitative proteomics strategy. *Nat. Biotechnol* **28**, 710-721 (2010).
11. Webb-Robertson, B.-J. M. *et al.* Review, evaluation, and discussion of the challenges of missing value imputation for mass spectrometry-based label-free global proteomics. *J. Proteome Res.* **14**, 1993-2001 (2015).
12. Fonville, J. M. *et al.* Robust Data Processing and Normalization Strategy for MALDI Mass Spectrometric Imaging. *Anal. Chem.* **84**, 1310-1319 (2012).
13. Tung, F., Wong, A. & Clausi, D. A. Enabling scalable spectral clustering for image segmentation. *Pattern Recognit.* **43**, 4069-4076 (2010).
14. Xiao, X. *et al.* Lymphotoxin β receptor-mediated NFκB signaling promotes glial lineage differentiation and inhibits neuronal lineage differentiation in mouse brain neural stem/progenitor cells. *J. Neuroinflammation* **15**, 49 (2018).
15. Rozenblum, G. T., Kaufman, T. & Vitullo, A. D. Myelin Basic Protein and a Multiple Sclerosis-related MBP-peptide Bind to Oligonucleotides. *Mol. Ther. Nucleic Acids* **3**, e192-e192 (2014).
16. Khodanovich, M. *et al.* Quantitative Imaging of White and Gray Matter Remyelination in the Cuprizone Demyelination Model Using the Macromolecular Proton Fraction. *Cells* **8** (2019).
17. Sharma, K. *et al.* Cell type– and brain region–resolved mouse brain proteome. *Nat. Neurosci.* **18**, 1819-1831 (2015).

18 Jung, S. Y. *et al.* An Anatomically Resolved Mouse Brain Proteome Reveals Parkinson Disease-relevant Pathways. *Mo.l Cell Proteomics* **16**, 581-593 (2017).
19 Tian, G. & Cowan, N. J. Tubulin-specific chaperones: components of a molecular machine that assembles the α/β heterodimer. *Methods Cell Biol.* **115**, 155-171 (2013).
20 Blanco, G. Na,K-ATPase Subunit Heterogeneity as a Mechanism for Tissue-Specific Ion Regulation. *Semin. Nephrol.* **25**, 292-303 (2005).
21 Lavoie, L., Levenson, R., Martin-Vasallo, P. & Klip, A. The Molar Ratios of α and β Subunits of the Na+−K+-ATPase Differ in Distinct Subcellular Membranes from Rat Skeletal Muscle. *Biochemistry* **36**, 7726-7732 (1997).
