# Supplementary material for: In-depth mapping of protein localizations in whole tissue by micro-scaffold assisted spatial proteomics (MASP)
Source: Nat Commun. 2022 Dec 14;13:7736. doi: 10.1038/s41467-022-35367-2 (PMC9751300; doi:10.1038/s41467-022-35367-2)
Supplement: Supplementary file 2 — Reporting Summary [file 41467_2022_35367_MOESM2_ESM.pdf]

nature portfolio

Corresponding author(s): Jun Qu

Last updated by author(s): Nov 10, 2022

Reporting Summary

Nature Portfolio wishes to improve the reproducibility of the work that we publish. This form provides structure for consistency and transparency in reporting. For further information on Nature Portfolio policies, see our Editorial Policies and the Editorial Policy Checklist.

Statistics

For all statistical analyses, confirm that the following items are present in the figure legend, table legend, main text, or Methods section.

n/a	Confirmed	
[ ]	[x]	The exact sample size (*n*) for each experimental group/condition, given as a discrete number and unit of measurement
[ ]	[x]	A statement on whether measurements were taken from distinct samples or whether the same sample was measured repeatedly
[ ]	[x]	The statistical test(s) used AND whether they are one- or two-sided<br>*Only common tests should be described solely by name; describe more complex techniques in the Methods section.*
[x]	[ ]	A description of all covariates tested
[x]	[ ]	A description of any assumptions or corrections, such as tests of normality and adjustment for multiple comparisons
[ ]	[x]	A full description of the statistical parameters including central tendency (e.g. means) or other basic estimates (e.g. regression coefficient) AND variation (e.g. standard deviation) or associated estimates of uncertainty (e.g. confidence intervals)
[ ]	[x]	For null hypothesis testing, the test statistic (e.g. *F*, *t*, *r*) with confidence intervals, effect sizes, degrees of freedom and *P* value noted<br>*Give P values as exact values whenever suitable.*
[x]	[ ]	For Bayesian analysis, information on the choice of priors and Markov chain Monte Carlo settings
[x]	[ ]	For hierarchical and complex designs, identification of the appropriate level for tests and full reporting of outcomes
[ ]	[x]	Estimates of effect sizes (e.g. Cohen's *d*, Pearson's *r*), indicating how they were calculated

Our web collection on statistics for biologists contains articles on many of the points above.

Software and code

Policy information about availability of computer code

Data collection	Thermo Scientific Xcalibur (Version 4.2.47, Thermo Fisher Scientific)
Data analysis	SIEVE, Version 2.2.58 SP2, (Thermo Fisher Scientific);<br>MS-GF+, Version 10089, (https://omics.pnl.gov/software/ms-gf);<br>IDPicker, Version 3.1.643.0 64-bit, (http://proteowizard.sourceforge.net/idpicker/);<br>R, Version 4.0.5 or higher, (https://www.r-project.org/);<br>R Studio, Version 2021.09.0-351 or higher, (https://www.rstudio.com/products/rstudio/download/);<br>R Shiny interactive web application package “UHR.IonStar”, Version 1.5, (https://github.com/JunQu-Lab/UHRIonStarApp);<br>R Shiny interactive web application package "MAsP", Version 1.0, (https://github.com/JunQu-Lab/MAsP);<br>Autodesk Fusion 360 software, Version 2.0.10806, (https://www.autodesk.com);<br>DAVID Functional Annotation, Version 6.8, (https://david.ncifcrf.gov/);<br>KEGG analysis, Version 101.0, (https://www.genome.jp/kegg/).

For manuscripts utilizing custom algorithms or software that are central to the research but not yet described in published literature, software must be made available to editors and reviewers. We strongly encourage code deposition in a community repository (e.g. GitHub). See the Nature Portfolio guidelines for submitting code & software for further information.

Data

Policy information about availability of data

All manuscripts must include a data availability statement. This statement should provide the following information, where applicable:

- Accession codes, unique identifiers, or web links for publicly available datasets
- A description of any restrictions on data availability
- For clinical datasets or third party data, please ensure that the statement adheres to our policy

All data presented in this manuscript are available as supplementary data files. The LC-MS raw data generated in this study have been deposited in the ProteomeXchange Consortium via the PRIDE partner repository with the dataset identifier PXD037041 [http://proteomecentral.proteomexchange.org/cgi/GetDataset?ID=PXD037041](MASP micro-specimen samples at different spatial locations). Uniprot-SwissProt mouse database (version downloaded on 07/13/2018, 16,961 entries)

Human research participants

Policy information about studies involving human research participants and Sex and Gender in Research.

Reporting on sex and gender	*Use the terms sex (biological attribute) and gender (shaped by social and cultural circumstances) carefully in order to avoid confusing both terms. Indicate if findings apply to only one sex or gender; describe whether sex and gender were considered in study design whether sex and/or gender was determined based on self-reporting or assigned and methods used. Provide in the source data disaggregated sex and gender data where this information has been collected, and consent has been obtained for sharing of individual-level data; provide overall numbers in this Reporting Summary. Please state if this information has not been collected. Report sex- and gender-based analyses where performed, justify reasons for lack of sex- and gender-based analysis.*
Population characteristics	*Describe the covariate-relevant population characteristics of the human research participants (e.g. age, genotypic information, past and current diagnosis and treatment categories). If you filled out the behavioural & social sciences study design questions and have nothing to add here, write "See above."*
Recruitment	*Describe how participants were recruited. Outline any potential self-selection bias or other biases that may be present and how these are likely to impact results.*
Ethics oversight	*Identify the organization(s) that approved the study protocol.*

Note that full information on the approval of the study protocol must also be provided in the manuscript.

Field-specific reporting

Please select the one below that is the best fit for your research. If you are not sure, read the appropriate sections before making your selection.

☒ Life sciences ☐ Behavioural & social sciences ☐ Ecological, evolutionary & environmental sciences

For a reference copy of the document with all sections, see nature.com/documents/nr-reporting-summary-flat.pdf

Life sciences study design

All studies must disclose on these points even when the disclosure is negative.

Sample size	No statistical methods were used to predetermine sample size because of the sample size of the current study was determined based on spatial resolution and the size of the mouse brain.
Data exclusions	No data were excluded.
Replication	The exact number of replication for all sample preparation optimization experiments was described in figure legends and our attempts at replication were successful. The quality control sample replicates for LC/MS analysis were performed multiple times and repeated 9 times with reproducible and similar results.
Randomization	The LC-MS measurement were randomized. For sample preparation optimization experiment, all the samples were collected from random locations of the mouse tissue and randomly allocated to different groups. For the spatial proteomic analysis, the randomization was not conducted as only one experiment group exists.
Blinding	For the spatial proteomic analysis, the investigator was not blinded to group allocation during data collection or analysis because only one group exists for the spatial experiment. For sample preparation optimization experiment, the investigator was not blinded to group allocation during data collection or analysis because it is not possible to blind the experimenter to the experimental conditions while still being physically present during the experiment.

Reporting for specific materials, systems and methods

We require information from authors about some types of materials, experimental systems and methods used in many studies. Here, indicate whether each material, system or method listed is relevant to your study. If you are not sure if a list item applies to your research, read the appropriate section before selecting a response.

Materials & experimental systems

n/a	Involved in the study
☒	☐ Antibodies
☒	☐ Eukaryotic cell lines
☒	☐ Palaeontology and archaeology
☐	☒ Animals and other organisms
☒	☐ Clinical data
☒	☐ Dual use research of concern

Methods

n/a	Involved in the study
☒	☐ ChIP-seq
☒	☐ Flow cytometry
☒	☐ MRI-based neuroimaging

Animals and other research organisms

Policy information about studies involving animals; ARRIVE guidelines recommended for reporting animal research, and Sex and Gender in Research

Laboratory animals	Male Swiss Webster mice (8-week-old) were group housed under a 12-h-light/dark cycle (8am-8pm) at constant temperature (25°C) and humidity (40-60%) with ad lib access to food and water. Animals arriving from external sources were allowed at least one week of habituation to housing conditions prior to experimentation.
Wild animals	No wild animals were used in the study.
Reporting on sex	The finding is only apply to one sex (male) of mice. The sex was considered during experiment design and the male mice were chosen because male mice are more widely used than female mice in research.
Field-collected samples	No field collected samples were used in the study.
Ethics oversight	All animal experiments were performed according to the protocols approved by the Roswell Park Institutional Animal Care and Use Committee (IACUC).

Note that full information on the approval of the study protocol must also be provided in the manuscript.
