# Supplementary material for: In-depth mapping of protein localizations in whole tissue by micro-scaffold assisted spatial proteomics (MASP)
Source: Nat Commun. 2022 Dec 14;13:7736. doi: 10.1038/s41467-022-35367-2 (PMC9751300; doi:10.1038/s41467-022-35367-2)
Supplement: Supplementary file 3 — Description of Additional Supplementary Files [file 41467_2022_35367_MOESM3_ESM.pdf]

Description of Additional Supplementary Files

Supplementary Movie 1.
Description: The workflow of the precisely-controlled pressurization uniformly across the tissue slide.

File Name: Supplementary Data 1.
Description: The log2 abundance of the 5,019 proteins quantified by MASP with spatial locations.

File Name: Supplementary Data 2.
Description: The spatial locations of the micro-specimens.

File Name: Supplementary Data 3.
Description: KEGG pathways of the 5019 proteins quantified by MASP.

File Name: Supplementary Data 4.
Description: Gene Ontology Biological Processes of the 5019 proteins quantified by MASP.
